# Van-mediated self-aggregating photothermal agents combined with multifunctional magnetic nickel oxide nanoparticles for precise elimination of bacterial infections

**DOI:** 10.1186/s12951-022-01535-1

**Published:** 2022-07-14

**Authors:** Ting Du, Jiangli Cao, Zehui Xiao, Jiaqi Liu, Lifei Wei, Chunqiao Li, Jingbo Jiao, Zhiyong Song, Jifeng Liu, Xinjun Du, Shuo Wang

**Affiliations:** 1 State Key Laboratory of Food Nutrition and SafetyKey Laboratory of Food Nutrition and Safety, Ministry of Education, College of Food Science and EngineeringCollege of Food Science and Engineering, Tianjin University of Science and Technology, Tianjin, 300457 People’s Republic of China; 2 Tianjin Key Laboratory of Food Science and Health, School of Medicine, Nankai University, Tianjin, 300071 People’s Republic of China; 3 College of Sicence, Huazhong Agricultural University, Wuhan, 430070 People’s Republic of China

**Keywords:** Self-aggregating AuNPs, Photothermal therapy, NiO NPs, Magnetic enrichment, Infected wound healing

## Abstract

Building a novel and efficient photothermal antibacterial nanoplatform is a promising strategy for precise bacterial elimination. Herein, a nanocomposite NiO NPs@AuNPs@Van (NAV) for selective MRSA removal was constructed by electrostatic self-assembly of highly photothermal magnetic NiO NPs and vancomycin (Van)-modified gold nanoparticles (AuNPs). In the presence of MRSA and under NIR irradiation, Van-mediated AuNPs can self-aggregate on MRSA surface, generating photothermal effect in situ and killing 99.6% MRSA in conjunction with magnetic NiO NPs. Additionally, the photothermal efficiency can be improved by magnetic enrichment due to the excellent magnetism of NAV, thereby enhancing the bactericidal effect at a lower experimental dose. In vitro antibacterial experiments and full-thickness skin wound healing test demonstrated that this combination therapy could effectively accelerate wound healing in MRSA-infected mice, increase collagen coverage, reduce IL-6 and TNF-α content, and upregulate VEGF expression. Biological safety experiments confirmed that NAV has good biocompatibility in vivo and in vitro. Overall, this work reveals a new type of nanocomposite with enhanced photothermal antibacterial activity as a potential nano-antibacterial agent for treating bacteria-infected wounds.

**Supplementary Information:**

The online version contains supplementary material available at 10.1186/s12951-022-01535-1.

## Introduction

A recent report indicated that around 700,000 people worldwide die each year from drug-resistant bacteria, and this number is expected to increase to 10 million by 2050, which will exceed the total number of deaths from cancer (8.2 million per year) [1]. Antibiotics are drugs currently used clinically to fight bacterial infections. However, traditional antibiotics are becoming less effective due to the emergence of drug-resistant (DR) or multi-drug resistant (MDR) bacterial strains [2, 3]. Methicillin-resistant *S. aureus* (MRSA) is the most typical drug-resistant pathogen, which can be transmitted between humans and animals, posing a serious threat to human health [4, 5]. MRSA can attack many organs and tissues and cause various diseases such as endocarditis, chronic osteomyelitis, pneumonia and bacteremia [6, 7]. MRSA can resist the effects of many common antibiotics through a variety of drug resistance mechanisms, such as thickening of cell wall, increase of cell membrane efflux pumps, mutation of cytoplasmic drug target, drug modifications and change of bacterial colonization status [8, 9]. In view of the more complex problem of the treatment of DR or MDR bacterial strains, the development of new approaches based on new treatment regimens with lower induction of drug resistance is becoming more and more urgent.

Thanks to the rapid development of nanotechnology, various nanomaterial-based antibacterial materials and strategies have been proposed, including chemotherapy, photocatalytic therapy, antibacterial peptides, photodynamic therapy, photothermal therapy (PTT) and so on [10–14]. Among them, near infrared (NIR) laser-triggered PTT is considered as an effective alternative to traditional antibacterial strategies because of its advantages of simplicity, deep tissue permeability, minimal invasiveness, low light damage, easy positioning, and non-drug resistance, particularly in treating infected wounds. Photothermal wound dressings usually contain NIR absorbers and kill microorganisms by denaturing bacterial proteins/enzymes, inhibiting their basic intracellular reactions, and inducing irreversible bacterial ablation through the conversion of light energy into heat to increase local temperature (> 50 °C) [15].

Recent studies have shown that there are four types of common photothermal materials: noble metal nanomaterials [16–18], carbon-based nanomaterials [19, 20], organic compound materials [21–23], and metallic/non-metallic compound nanomaterials [24, 25]. Carbon based nanomaterials mainly refer to graphene oxide and carbon nanotubes, which are characterized by good thermal stability and high drug loading capacity, making them a good carrier for photothermal therapy and chemotherapy. However, the preparation of such materials is relatively difficult, and the light absorption coefficient is low in the near-infrared region [26]. Organic compound nanomaterials include small molecular dyes (such as indocyanine green, prussian blue and thiadiazole derivatives [27–29]) and supramolecular complexes (such as porphyrin rings, conjugated polymers, polyaniline, polypyrrole and polythiophene [30–32]). The significant advantage of this type of material is its high biocompatibility, but it is less stable under light and easy to photodegrade. Metal and non-metal compound nanomaterials are metal sulfides, such as molybdenum disulfide (MoS_2_) [33] and copper sulfide (CuS) [34]. Metal sulfides have been used to inhibit bacteria, but the preparation process of most materials is complex and difficult for large-scale production.

Noble metal photothermal nanomaterials, such as gold, silver, platinum, etc., can absorb light energy of specific wavelengths and generate oscillating free electrons on the surface [35]. Among them, Au nanoparticles have become a promising photothermal agent due to their good light stability, low toxicity, and easy surface modification [36]. Moreover, the shape of Au nanoparticles can be adjusted for strong NIR absorption, which can finally be transferred to the surrounding environment in the form of heat [37]. For instance, Han et al. reported a functional Au nanostar as a photothermal agent for clearing bacterial infections [2]. Youn et al. successfully developed the rabies virus glycoprotein (RVG) peptide-PEG-capped silica-gold nanorods (RVG-PEG-AuNRs@SiO_2_), which can respond to NIR laser irradiation to induce hyperthermia and effectively inhibit brain tumors in mice [38]. You et al. proposed a specific photothermal ablation therapy based on TNYL peptide conjugated hollow gold nanospheres (TNYL-HAuNS), which can significantly inhibit the growth of endometriosis and induce ectopic endometrial atrophy and degeneration [39]. Richter et al. reported a scaffold composed of electrospun Jellyfish-based nanofibers and decorated with Au–Ag bimetallic nanoparticles, which was demonstrated to be able to disrupt and eliminate the regeneration of mature biofilm colonies successfully [40]. Nevertheless, single-mode PTT usually has the disadvantage of prolonged exposure to high-power-density NIR lasers, which may lead to inflammation and thermal damage of nearby healthy tissues [41, 42]. Additionally, the non-specific dispersion of plasma-absorbing nanomaterials on the bacterial surface cannot ensure the complete transfer of the hyperthermia converted from the absorbed light energy to the bacteria under NIR laser irradiation. Therefore, it is imperative to design a treatment model for accurate attack on bacteria-infected tissues without damaging adjacent normal tissues.

**Scheme 1:**
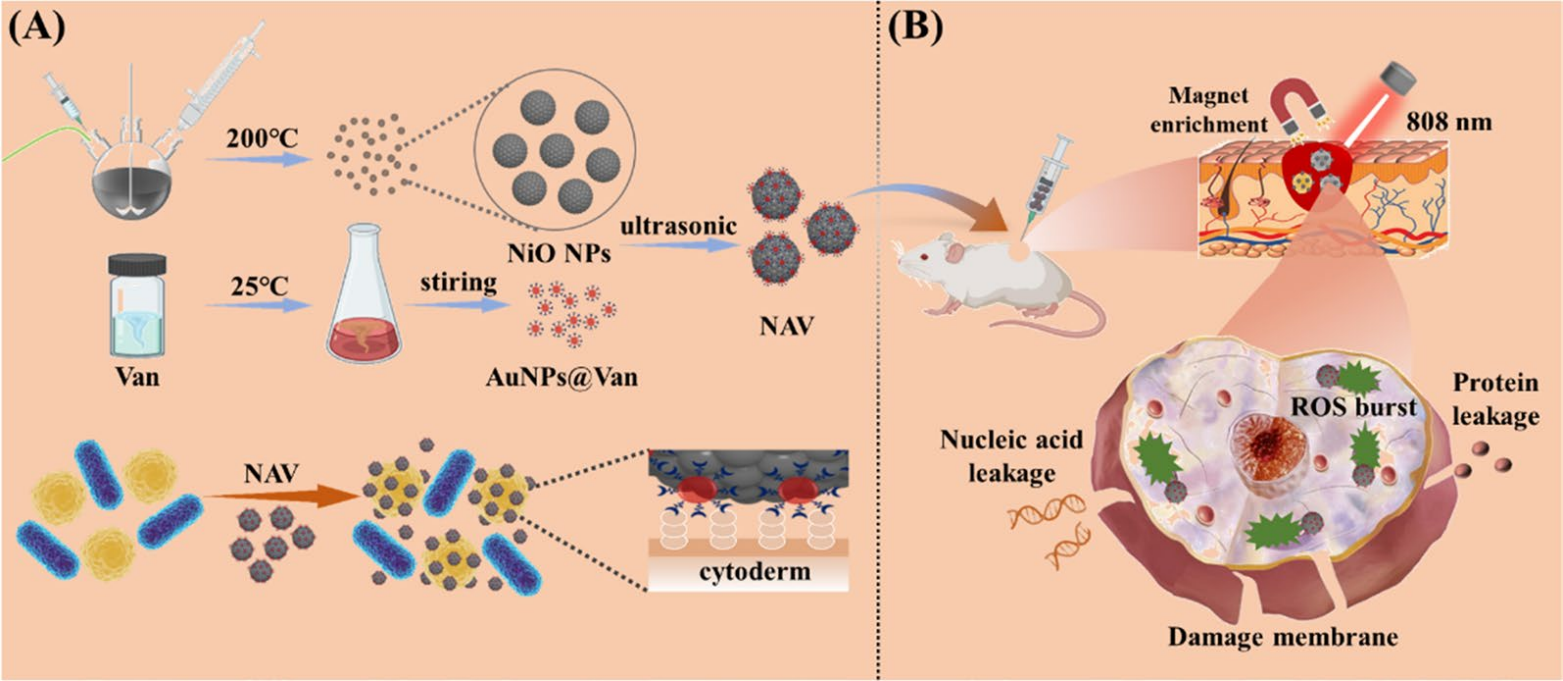
**A** Schematic diagram for NAV preparation; NAV can preferentially accumulate on MRSA surface under Van mediation. **B** In situ photothermal and magnetic enrichment of bacteria-infected tissues stimulated by NIR irradiation and its application in eliminating bacteria in vivo

Inspired by this idea and based on aforementioned reports, we proposed an accumulation strategy using MRSA as a template for selective removal of bacterial infections while protecting adjacent normal tissues. The targeted accumulation of nanomaterials on MRSA was achieved by modifying spherical Au nanoparticles with Van (AuNPs@Van), which was bonded to D-Ala-D-Ala part of the Gram-positive bacterial cell wall and anchored on the bacterial surface. AuNPs@Van was expected to accumulate on the MRSA surface and reach a concentration to activate the plasma coupling effect between particles (Scheme 1A). Subsequently, the AuNPs and magnetic NiO NPs were electrostatically self-assembled to form NAV nanocomposites. Nickel oxide nanoparticles possess good chemical stability and magnetism, and they were reported to have the ability to capture and bind bacteria [43]. However, to our knowledge, no study has been performed on their photothermal conversion ability and magnetic enrichment performance in biomedicine. A large number of studies have pointed out that PTT-assisted multi-modal collaborative treatment is the most effective strategy to solve single-mode defects due to the combination of different advantages in a single-mode method, which can shorten the NIR time and improve the antibacterial efficiency [44, 45].

In this study, NiO NPs were discovered to have excellent photothermal properties. The introduction of NiO NPs into the nanocomposite system we prepared could greatly shorten the irradiation time and improve the efficiency of photothermal sterilization. Meanwhile, under the action of an external magnetic field, the photothermal efficiency was further improved through magnetic enrichment, and a remarkable sterilization effect could be achieved at a lower experimental dose, thereby improving biological safety. Overall, the hyperthermia of NAV was triggered in situ in the infected tissue under Van mediation and NIR irradiation, thereby achieving selective and effective bacterial removal (Scheme 1B).

## Materials and methods

### Materials and reagents

The materials used in this study included diethylene glycol (DEG, ≥ 99.0%) from Sigma-Aldrich Co., Ltd (Shanghai, China); nickel (II) chloride (NiCl_2_, ≥ 99.0%) from Macklin Reagent Co. (Shanghai, China); chloroauric acid (HAuCl_4_, ≥ 99.0%) and vancomycin HCl (Van) from Aladdin (Shanghai, China); thiol-PEG-amine (NH_2_-PEG-SH; MW: 2000 Da) from Solarbio Sci-Tech Co. (Beijing, China); N-hydroxysuccinimide (NHS) from Shanghai Yuanye Bio-technology Co., Ltd. (Shanghai, China); 1-(3-Dimethylaminopropyl)-3-ethylcarbodiimide hydrochloride (EDC) from Beyotime (Beijing, China); sodium citrate (NaCit, ≥ 99.0%) form Victor Chemical Products Trading Co., LTD. (Tianjin, China); NaOH (≥ 96.0%) from Tianjin Jiangtian Chemical Technology Co., LTD. (Tianjin, China). The gram-positive MRSA (800,926) were provided by our laboratory. All experiments used Milli-Q grade water (18.2 MΩ).

### Synthesis of magnetic nickel oxide nanoparticles (NiO NPs)

The NiO NPs were synthesized based on a modified previously reported thermal hydrolysis method [43]. Briefly, 15 mM of NaOH was added to 6.0 mL of DEG solution and stirred at 150 °C until the solution was slightly yellow, and the resultant mixture was named NaOH/DEG. Next, 4.0 mM NiCl_2_, 1.7 mM NaCit and 20 mL DEG were mixed in a 250 mL three-neck round bottom flask in N_2_ atmosphere, followed by heating to 200 °C and stirring at 200 rpm/min for 30 min. Subsequently, the obtained NaOH/DEG was quickly injected into the above round bottom flask, and the color of the system was seen to change from green to black. After 2 h of reaction, the mixture was naturally cooled to room temperature to obtain NiO NPs. After two washes with ethanol and three washes with deionized water, the NiO NPs were stored in absolute ethanol for further use.

### Synthesis of AuNPs@Van nanoparticles

The synthesis of AuNPs@Van NPs followed two steps. First, 250 μL HAuCl_4_ (0.1 M) was mixed with 100 mL of deionized water, followed by fully stirring, heating to boiling, quickly adding 2.5 mL of 1% NaCit, and further heating for 20 min to obtain AuNPs. After cooling to room temperature, the AuNPs were stored at 4 °C for later use.

In the second step, 10 mg of Van was dissolved in 1.0 mL of deionized water, followed by adding EDC (50 μL, 50 mg/mL) and NHS (50 μL, 25 mg/mL) for 1 h of stirring reaction to activate Van. Next, the activated Van was mixed with NH_2_-PEG-SH (MW: 2000 Da) and slowly shaken at room temperature for 8 h to thiolate the Van, followed by adding the above prepared AuNPs (75 mL) and incubation for another 8 h to form Au–S bonds between Van and AuNPs. Subsequently, the resulting solution (AuNPs@Van) was transferred to a dialysis bag (molecular weight cutoff, 35 kDa) and washed several times with PBS buffer to remove excess Van. Finally, the purified AuNPs@Van NPs were stored at 4 °C for further use. The load of Van on AuNPs@Van was calculated according to the standard absorption curve of Van.

### Synthesis of NiO NPs@AuNPs@Van (NAV)

Briefly, NiO NPs (1.0 mg/mL) were mixed with AuNPs@Van (3.0 mg/mL), followed by ultrasonicating the mixture solution for 2 h to obtain NAV (self-assembly by electrostatic interaction). Based on the magnetic adsorption effect of NiO NPs, the obtained NAV nanoparticles are magnetically separated with a magnet and washed several times with deionized water to remove excess AuNPs@Van.

### Photothermal property of NAV

The photothermal conversion characteristics of NAV were investigated by measuring the temperature changes of NAV samples under 808 nm laser irradiation. Briefly, different concentrations of NAV in an EP tube were exposed to laser light (808 nm, 1.8 W/cm^2^) for 10 min, and PBS group was set as the control group. The FLUKE Ti400 + infrared thermal camera was used to record the real-time temperature value of the NAV and the corresponding thermal image every two minutes. The NAV thermal stability was measured during 5 laser on/off cycles. The photothermal conversion efficiency of NAV was measured as previously reported [46].

### In vitro antibacterial experiment

The in vitro antibacterial activity of NAV against MRSA was evaluated by the minimum inhibitory concentration (MIC) and the spread plate method. The MIC was estimated by the two-fold dilution method. The bacterial suspension (100 μL, 10^7^ CFU/mL) and 100 μL NAV of different concentrations (250, 125, 62.5, 31.3, 15.6, 7.80 and 0 μg/mL) were pre-mixed in a 96-well plate and incubated for 4–5 h. At the time when the control group was cultured to a turbid state, and the NAV treatment group showed no turbidity visible to the naked eye and an OD value of less than 0.1, the corresponding concentration was defined as the MIC.

For the spread plate method, bacterial suspensions (10^3^ CFU/mL) were co-incubated with different concentrations of NAV (250, 125, 62.5, 31.3, 15.6, 7.80 and 0 μg/mL) for 1 h at 37 °C, followed by exposing or unexposing the NAV-bacteria conjugate to 808 nm NIR laser irradiation (NAV-NIR_(+)_ and NAV-NIR_(−)_) for 10 min. Bacterial suspensions cultured in PBS with and without NIR irradiation were used as control groups (PBS-NIR_(+)_ and PBS-NIR_(−)_). Afterward, 50 μL of the bacterial solution was evenly coated on a fresh LB agar plate and cultured at 37 °C for 24 h. The number of colonies formed was recorded and the survival rate of the bacteria was computed. The same method was applied to assess the antibacterial activity of different concentrations of NiO NPs and AuNPs@Van.

### Live/dead staining assay

A live/death staining assay was applied to study the effect of NAV on bacterial viability. Specifically, bacterial suspensions (10^7^ CFU/mL) were treated with PBS (PBS-NIR_(+)_ and PBS-NIR_(-)_) and NAV (NAV-NIR_(+)_ and NAV-NIR_(-)_), followed by washing with sterile water and staining with DAPI and PI for 15 min in the dark. After rinsing several times with sterile water, the cells were dropped onto the glass slide, and the living/ dead bacteria were visualized via an Olympus IX73 microscope.

### Morphological observation of bacteria

Changes in the bacterial morphologies were recorded by TEM and SEM imaging. Briefly, after various treatments, the bacterial suspensions (10^7^ CFU/mL) were rinsed three times with sterile water and then fixed with glutaraldehyde (2.5%) overnight. After washing with sterile water, all samples were dehydrated separately with 30%, 50%, 70%, 80%, 90% and 100% ethanol for 10 min. Next, 10 μL dehydrated bacterial suspension was dropped onto silica wafer or copper mesh. After air drying, all samples were imaged by a SEM (FEI_Apreo) and TEM (talos G2 200X).

### Detection of reactive oxygen species (ROS) generation

ROS indicator DCFH-DA dye was used to detect ROS production in bacterial cells after various treatments (PBS-NIR_(−)_, PBS-NIR_(+)_, NAV-NIR_(−)_ and NAV-NIR_(+)_). The NAV-bacteria conjugates in different treatment groups were cultured with DCFH-DA (10 μM) for 1 h at 37 °C in the dark, followed centrifugation and rinsing with PBS. Finally, the cell fluorescence intensity was examined and visualized through an Olympus IX73 microscope.

### Detection of protein and nucleic acid leakage

When the bacterial membrane is damaged, the cell components such as proteins, nucleic acids, electrolytes, etc. will leak. After various treatments, all samples were divided into 2 parts. One part was centrifuged to remove the supernatant, and the protein content was determined using the BCA protein concentration determination kit. For the other part of samples, the bacterial cell suspension was filtered with a 0.22 μm syringe filter and 200 μL filtrate was placed in a 96-well plate to measure the OD_260 nm_ value using a microplate reader (Waltham, MA, USA).

### Detection of ATP enzymatic activity

The steps for treating bacteria are the same as the aforementioned in vitro antibacterial test. The NAV-bacteria conjugate was rinsed three times with sterile water, followed by centrifugation to remove the supernatant and resuspension in PBS. After cell wall breaking treatment of the bacterial suspension, the total ATPase activity was analyzed for each group using the ultra-micro total ATPase test kit (Jiancheng Biotechnology Co., Nanjing, China). Finally, the absorbance value at 636 nm was investigated by a microplate reader (Waltham, MA, USA), and the total ATPase activity was calculated.

### In vitro cytotoxicity assay of NAV

MTT test was applied to evaluate the in vitro cytotoxicity of NAV at different concentrations (250, 125, 62.5, 31.3, 15.6, 7.80 and 0 μg/mL), using African Green Monkey Kidney (Vero) cells and mouse embryonic fibroblasts (NIH 3T3) cells as test model. Briefly, Vero cells or NIH 3T3 cells were seeded on a 96-well plate at a density of 2 × 10^5^ cells/well and cultured in a 37 °C/5% CO_2_ incubator until monolayer confluency. After discarding the inoculation solution, various concentrations of NAV were added to continue the culture for 24 and 48 h, followed by washing the cells with PBS and adding 20 μL/well of MTT solution (5.0 mg/mL) to continue the culture for 4 h. After discarding the supernatant, 150 μL DMSO was added to each well, and the absorbance at 490 nm was detected via a microplate reader (Waltham, MA, USA).

### Hemolytic assay of NAV

Hemolysis test was performed with the fresh blood of BALB/c mice [47]. The red blood cells (RBC) were collected by centrifugation and washed with PBS (0.02 M, pH = 7.4) until the supernatant appeared colorless. Next, the prepared RBCs were tenfold diluted to prepare the stock solution, followed by mixing the RBCs stock dispersion (0.2 mL) gently with different concentrations (250, 125, 62.5, and 31.3 μg/mL) of NAV (0.8 mL). Meanwhile, the RBCs stock dispersions mixed with deionized water and PBS were used as the positive and negative control, respectively. All samples were incubated at 37 °C for 30 min, followed by centrifuging the supernatant at 3000 rpm for 5 min, and measuring the absorbance of the supernatant at 545 nm through a microplate reader (Waltham, MA, USA). The hemolysis rate was calculated by the following formula:

Hemolysis ratio (%) = (A_S_−A_NC_)/(A_PC_−A_NC_) × 100% where A_S_, A_NC_ and A_PC_ represent the absorbance values of the sample, negative control and positive control, respectively.

### Animal experiment

To further evaluate the antibacterial activity of NAV in vivo, a mouse wound infection model was established. Female BALB/c mice (6–8 weeks old, six mice per group) were provided by SPF Biotechnology Co., Ltd. (Beijing, China). First, a wound with a diameter of about 8.0 mm was made on the back of each mouse under sterile conditions, and MRSA suspension (10^7^ CFU/mL, 100 μL) was injected subcutaneously into the mouse back. At 24 h post MRSA infection, the mice were randomly divided into 4 groups: I (PBS-NIR_(−)_), II (PBS-NIR_(+)_), III (NAV-NIR_(−)_) and IV (NAV-NIR_(+)_). The PBS-NIR_(−)_ group and NAV-NIR_(−)_ group were treated with 20 μL of PBS and NAV (125 μg/mL), respectively. The wounds of mice in the PBS-NIR_(+)_ group and NAV-NIR_(+)_ group were first smeared with PBS and NVA (125 μg/mL), followed by 1 min of 808 nm NIR laser irradiation (1.8 W/cm^2^). The wound temperature before and after NIR radiation was monitored using a thermal imaging camera. On day 0, 1, 3, 5, 7 and 9 of treatment, the mice were photographed, their body weight was recorded, and the wounds were dipped with sterile cotton swabs for colony counting. After 9 days of treatment, all mice were killed by cervical dislocation, and the main organs (kidney, heart, spleen, lung, liver) and wound skin tissues were collected. The wound skin tissues and major organs were immersed in 4% paraformaldehyde solution, embedded in paraffin, and sliced for H&E and Masson staining analysis. All the animal experiments were approved by the animal care and experiment committee of Tianjin University of Science & Technology (1140050032445).

### Statistical analysis

All tests were performed at least three times independently. The results were statistically analyzed by using one-way analysis of variance (ANOVA) in Graph Pad or Origin software. The *p* values < 0.05, < 0.01, < 0.001 and 0.0001 are indicated by the symbols of *, **, *** and ****, respectively.

## Results and discussion

### Synthesis and characterization of NAV

**Fig. 1:**
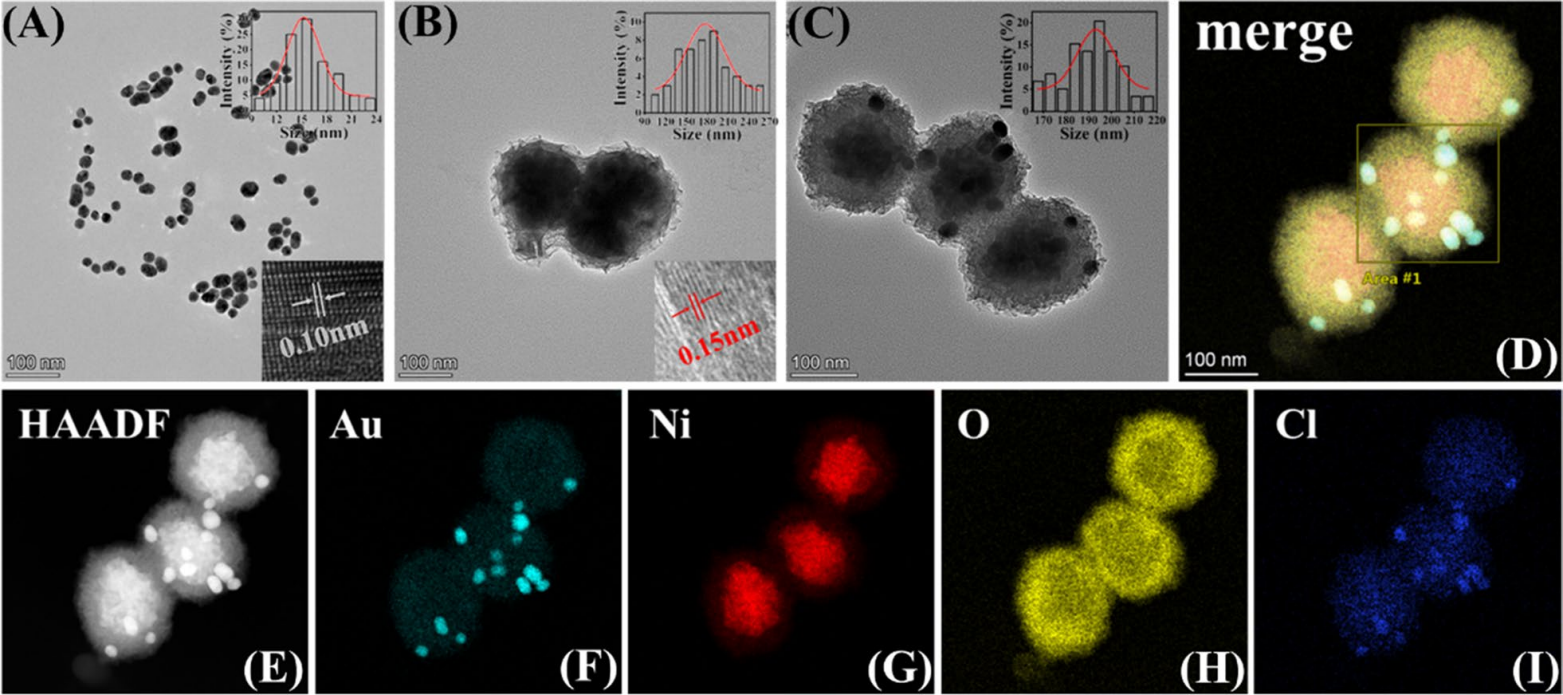
TEM images of **A** AuNPs@Van, **B** NiO NPs, and **C** NAV (see details in the text). **D**, **E** Scanning TEM images of NAV and elemental mapping images of **F** Au, **G** Ni, **H** O, and **I** Cl. Scale bar: 100 nm

In this study, we first synthesized AuNPs@Van and NiO NPs, followed by preparing NAV through electrostatic self-assembly. The morphologies of the prepared AuNPs@Van, NiO NPs and NAV were characterized by transmission electron microscope (TEM) imaging. In Fig. 1A, AuNPs@Van was seen to have a uniform spherical structure with an average diameter of 15.26 ± 0.22 nm as well as the lattice spacing of 0.10 nm (the inset image in the lower right corner). The synthesis of AuNPs@Van was based on the covalent coupling of the carboxyl group of Van with the amino group of NH_2_-PEG-SH activated by EDC and NHS, followed by Van modification with sulfhydryl group (-SH). When encountering AuNPs solution, strong Au–S bonds will be formed between Van and AuNPs. In Fig. 1I, the TEM element mapping showed the presence of Cl element (a typical element of Van) on the AuNPs surface, indicating the successful modification of Van. Figure 1B displays the morphology of the prepared NiO NPs, with an average diameter of 177.45 ± 5.43 nm calculated by randomly choosing 50 particles as well as a lattice spacing of 0.15 nm in the inset HRTEM image at the lower right bottom corner. Figure 1C shows the morphology of NAV, with AuNPs@Van being uniformly distributed on the surface of NiO NPs, and the size of NAV was 193.08 ± 1.61 nm, indicating the successful self-assembly of AuNPs@Van and NiO NPs. The uniform distribution of Au, Ni, O and Cl elements also indicated the successful loading of AuNPs@Van on NiO NPs, which can be confirmed by the HRTEM elemental mapping (Fig. 1D–I) and energy dispersive spectroscopy (EDS) (Additional file 1: Fig. S1).

**Fig. 2:**
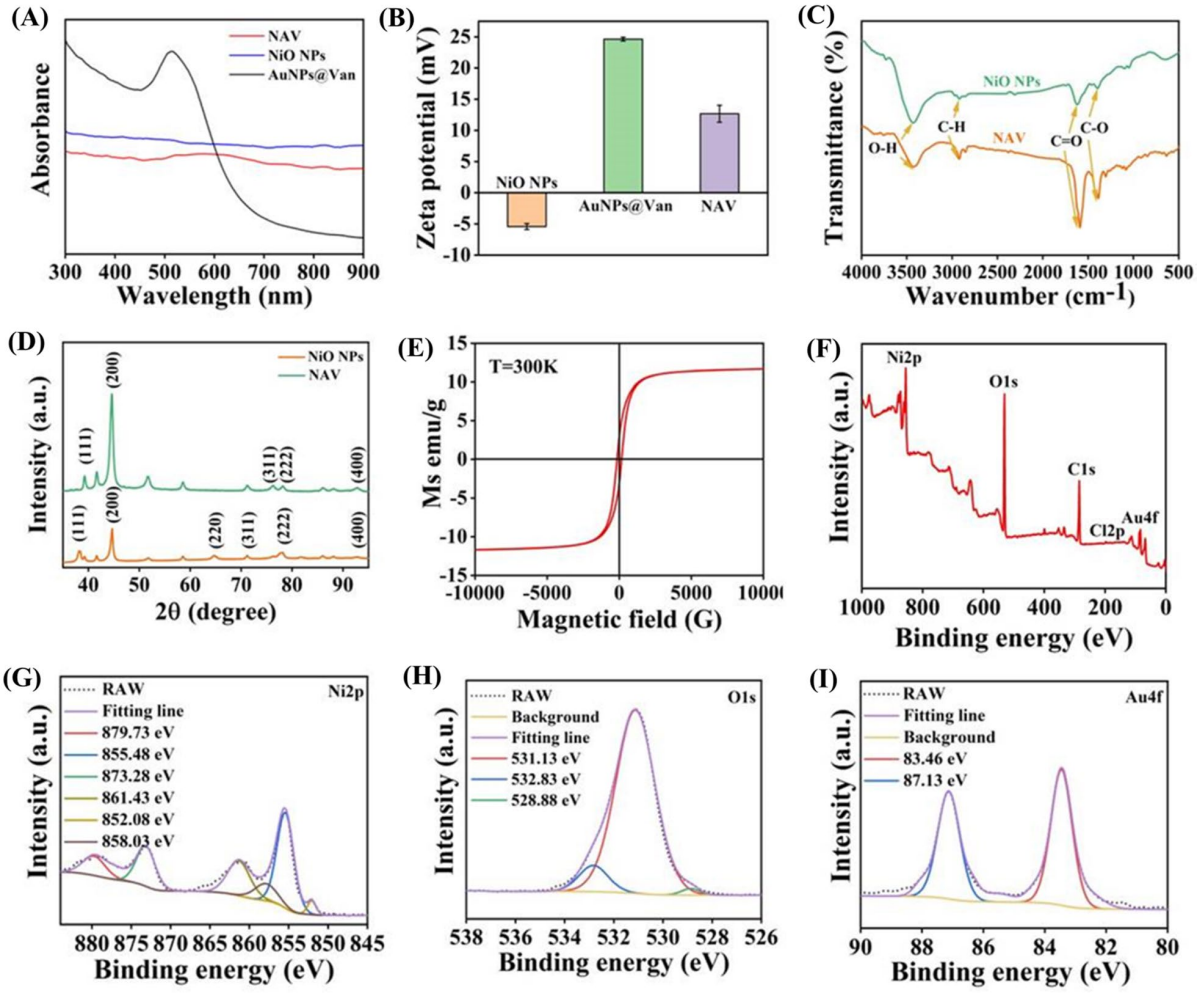
**A** UV–vis absorption spectra and **B** Zeta potential of AuNPs@Van, NiO NPs and NAV. **C** FT-IR spectra and **D** XRD patterns of NiO NPs and NAV. **E** Magnetic hysteresis loop of NAV. **F** XPS survey spectra and high-resolution XPS spectra of **G** Ni 2p, **H** O 1 s, and **I** Au 4f orbits for NAV

The chemical structure, composition and other properties of these samples were further characterized by UV–vis spectroscopy, zeta potential, magnetic hysteresis loop, X-ray diffraction (XRD) spectroscopy, Fourier-transform infrared spectroscopy (FTIR) and X-ray photoelectron spectroscopy (XPS). As illustrated in Fig. 2A, the absorption peak at 512 nm was the typical absorption peak of AuNPs@Van, and the UV–vis spectrum of NAV presented an obvious red shift and a wider absorption at 500–700 nm, proving the successful loading of AuNPs@Van on NiO NPs. The zeta potential values of AuNPs@Van, NiO NPs and NAV were 24.63 ± 0.31, -5.42 ± 0.47 and 12.67 ± 1.36 mV, respectively, demonstrating the successful preparation of NAV (Fig. 2B). NAV was almost identical to NiO NPs in the FTIR spectrum (Fig. 2C), with the characteristic absorption peaks at 3423, 2933, 1620, and 1386 cm^−1^ attributed to the stretching vibrations of O–H, C-H, C = O and C–O, respectively. Figure 2D displays the diffraction peaks of NiO NPs and NAV, with the peaks of NiO NPs at 39.3°, 44.6°, 76.3°, 78.1° and 92.0° corresponding to the (111), (200), (311), (222) and (400) crystal planes, respectively, which were closest to the standard document no. 47–1049 of the Joint Committee on Powder Diffraction. The significant increase of the Au diffraction peak intensity in the NAV XRD spectrum (64.6°, (220)) confirmed the successful loading of AuNPs@Van. In Fig. 2E, magnetic hysteresis loop analysis showed that NAV had a magnetization saturation (Ms) value of 11.9 emu/g. When the external magnetic field was completely removed, part of the residual magnetism was still preserved, indicating that NAV is a strong magnetic material [48]. Additionally, the elements and chemical states of NAV were measured via XPS, and in Fig. 2F, NAV was seen to mainly include Ni, O, C, Cl and Au. The presence of Cl also proved the successful modification of Van on AuNPs, further supporting the conclusion of TEM. The six intense peaks at 879.73, 855.48, 873.28, 861.43, 852.08 and 858.03 eV were ascribed to the binding energy of Ni 2p sat, Ni 2p 3/2(b), Ni 2p 1/2(b), Ni 2p sat, Ni 2p 3/2(a), and Ni 2p 1/2(a), respectively, which are consistent with those reported in the literature [43]. The typical peaks of O 1 s at 531.13, 532.83 and 528.88 eV were derived from NaCit and DEG (Fig. 2H). In Fg. 2I, the binding energy was seen to peak at 83.46 eV for Au 4f 7/2 and at 87.13 eV for Au 4f 5/2 [49]. These data revealed the successful modification of Van on AuNPs and confirmed the successful synthesis of NAV with strong magnetism.

### Photothermal effect of NAV

**Fig. 3:**
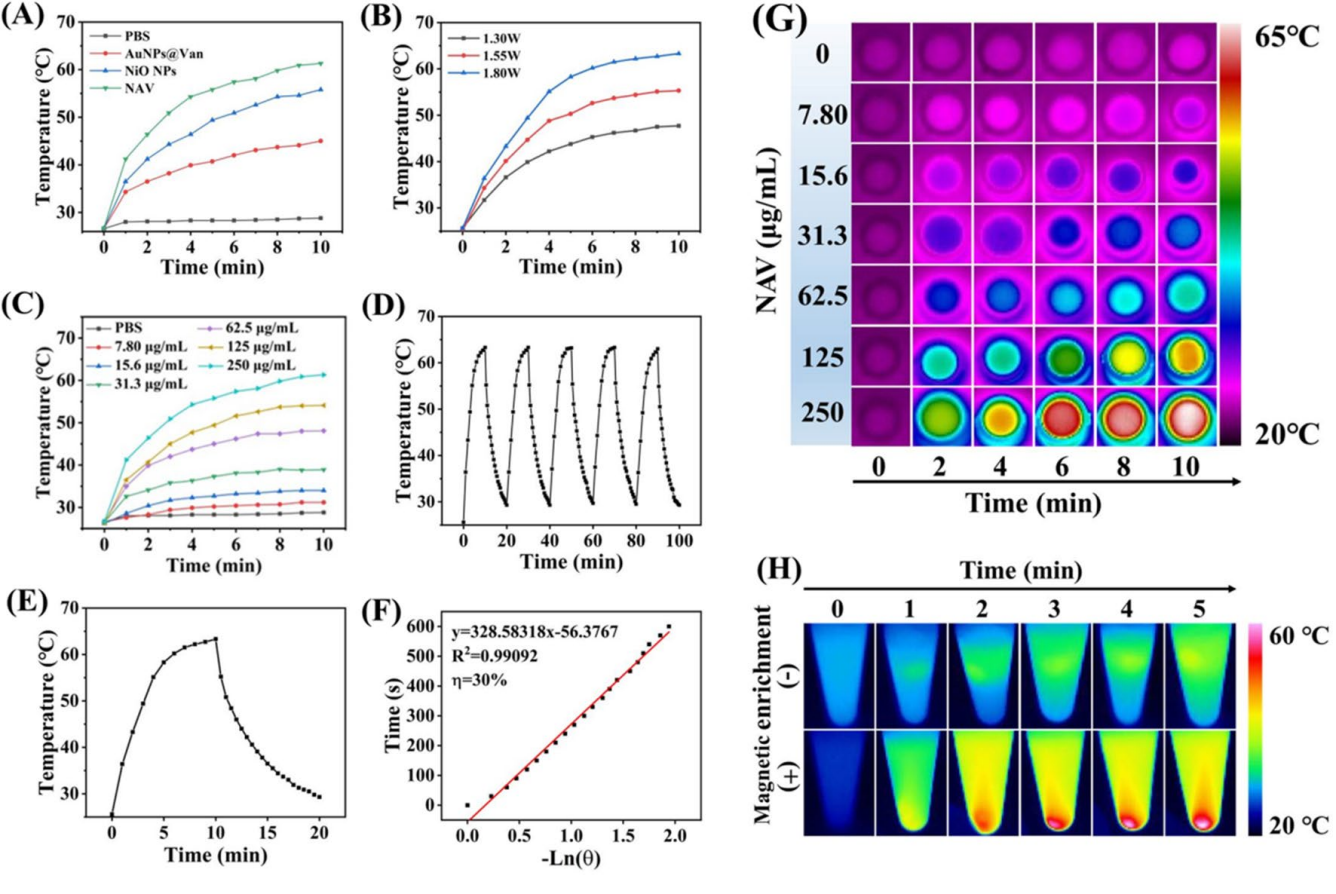
Photothermal effect of NAV. **A** Photothermal curves of PBS, AuNPs@Van, NiO NPs and NAV (NPs concentration: 125 μg/mL, 808 nm laser, 1.8 W/cm^2^, 10 min). **B** Photothermal curves of NAV (125 μg/mL) irradiated at different power laser densities (1.30, 1.55 and 1.80 W/cm^2^) for 10 min. **C** Photothermal curves of different concentrations of NAV (0, 7.80, 15.6, 31.3, 62.5, 125 and 250 μg/mL) at 1.8 W/cm^2^ for 10 min. **D** Heating and cooling profiles of NAV for 5 on/off cycles. **E** The heating/cooling curve of NAV under NIR laser irradiation. **F** The relationship between cooling time and −ln(θ) obtained from the results in **E**. **G** Infrared thermal imaging of NAV at different concentrations and different irradiation time intervals corresponding to **C**. **H** Photothermal images of NAV (125 μg/mL) within 5 min with and without magnetic enrichment

The photothermal effect of NAV was studied under 808 nm laser irradiation. The NAV UV–vis spectrum showed extensive absorption in the range of 500–700 nm (Fig. 2A), demonstrating the good photothermal conversion potential of NAV. As illustrated in Fig. 3A, under 808 nm laser irradiation (1.8 W/cm^2^), the NAV temperature increased from 26.6 to 61.3 °C, higher than that of PBS (from 26.6 to 28.8 °C), with the temperature of AuNPs@Van and NiO NPs increased from 26.6 °C to 45.0 °C and 55.8 °C, respectively. Surprisingly, we discovered for the first time that magnetic NiO NPs had photothermal conversion efficiency, and loading AuNPs@Van on its surface could improve the NAV photothermal conversion efficiency. As displayed in Fig. 3B and C, the temperature rise of NAV solution was positively correlated with NAV concentration, irradiation power, and irradiation time, indicating that NAV temperature can be precisely regulated by simply adjusting their values. In this study, we chose 1.8 W/cm^2^ as the lighting power, and the heating and cooling curves in Fig. 3D suggested that NAV had excellent photothermal stability even after 5 cycles of laser irradiation. Based on Fig. 3E and F, the NAV photothermal conversion efficiency was calculated to be 30%, which was higher than the values of the reported hollow Bi_2_S_3_ microspheres (23.8%) [50], CuS NPs (16.3%) [51] and iodine–starch-alginate hydrogel (17.2%) [52]. Figure 3G presents the infrared images of temperature changes over irradiation time for different concentrations of NAV monitored by a thermal imaging camera. All these results verified that NAV can be used as an effective photothermal agent for PTT.

Considering its excellent magnetic properties, the photothermal effect of NAV was further investigated under magnetic enrichment. In the case of no magnetic enrichment, the NAV temperature at 125 μg/mL showed only an increase of 4.2 °C after 5 min of NIR irradiation (Fig. 3H). Encouragingly, when associated with magnet enrichment, the NAV temperature rose from 25.6 to 53.4 °C after 5 min of NIR irradiation, indicating that magnetic enrichment could greatly improve the photothermal effect of NAV, thus decreasing the concentration of NAV as a photothermal agent, and increasing biological safety. Moreover, NiO NPs can capture bacteria by binding to the amino groups on the surface of the bacteria [43], inferring that under the action of magnetism, bacteria will be enriched, and the photothermal sterilization efficiency of NAV can be significantly improved. All these characteristics of NAV indicated its potential as an ideal photothermal scavenger of bacteria.

### In vitro antibacterial activity of NAV

**Fig. 4:**
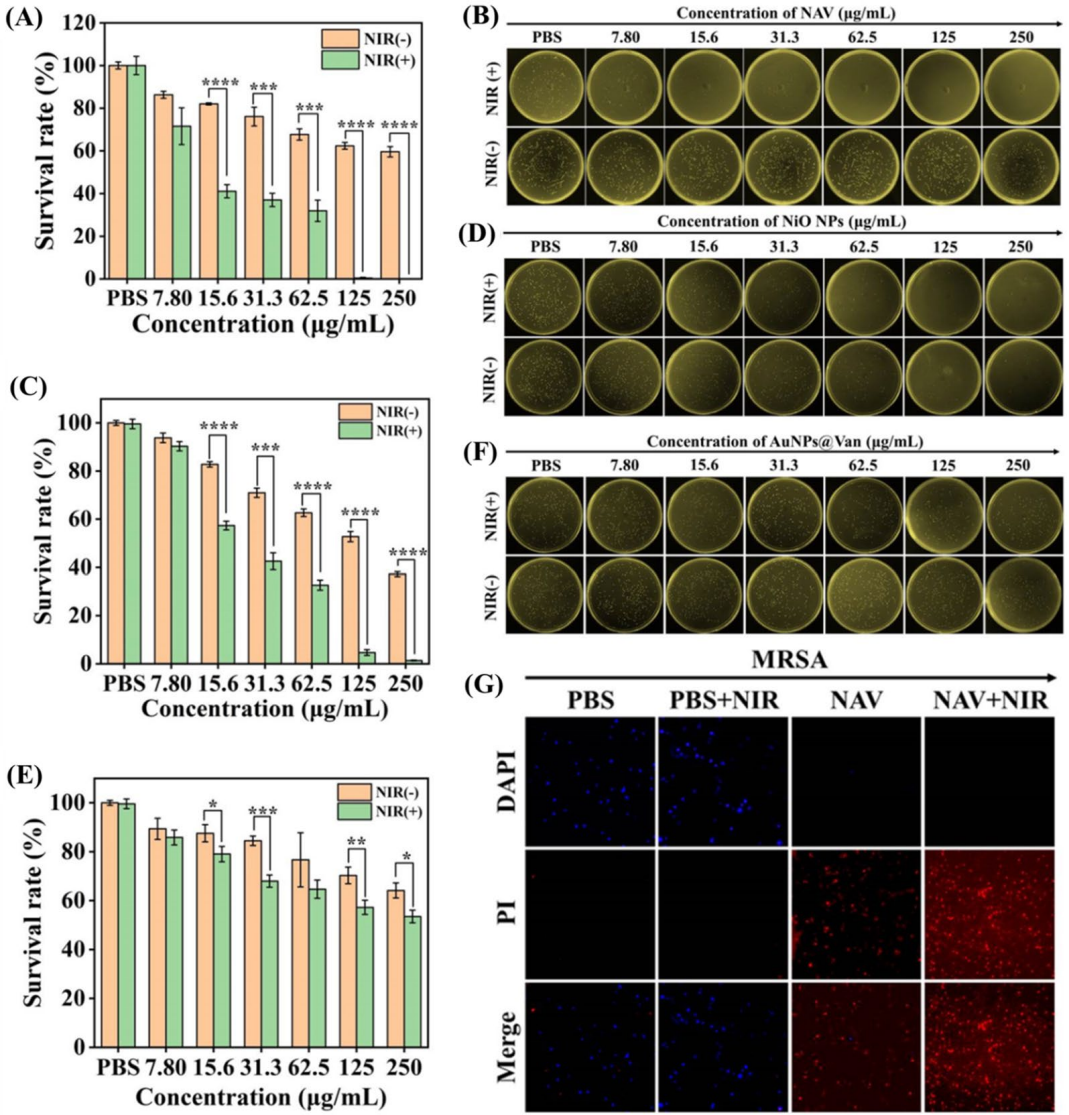
**A** The survival rate of MRSA after treatment with PBS (PBS-NIR_(-)_ and PBS-NIR_(+)_) and different concentrations (7.80, 15.6, 31.3, 62.5, 125 and 250 μg/mL) of NAV (NAV-NIR_(−)_ and NAV-NIR_(+)_). **B** Photographs of colony formation of MRSA corresponding to **A**. **C** The survival rate of MRSA after treatment with PBS (PBS-NIR_(−)_ and PBS-NIR_(+)_) and different concentrations of NiO NPs (NiO NPs-NIR_(−)_ and NiO NPs-NIR_(+)_). **D** Photographs of colony formation of MRSA corresponding to **C**. **E** The survival rate of MRSA after treatment with PBS (PBS-NIR_(−)_ and PBS-NIR_(+)_) and different concentrations of AuNPs@Van (AuNPs@Van-NIR_(−)_ and AuNPs@Van-NIR_(+)_). (**F**) Photographs of colony formation of MRSA corresponding to **E**. **G** Fluorescence staining images of MRSA in different treatment groups (PBS-NIR_(−)_, PBS-NIR_(+)_, NAV-NIR_(−)_ and NAV-NIR_(+)_), with live bacteria marked as blue by DAPI and dead bacteria marked as red by PI

The antibacterial capabilities of NAV, NiO NPs and AuNPs@Van against MRSA were investigated by measuring the minimum inhibitory concentration (MIC) and agar plate count assay. In Fig. 4A and B, a comparable number of viable colonies could be observed on the LB agar plates of the PBS-NIR_(−)_ group and PBS-NIR_(+)_ group, demonstrating that NIR irradiation will not affect the growth of MRSA. In Fig. 4C and D, the NAV-NIR_(–)_ group was seen to have weak antibacterial effect, with 37.7% reduction in the number of colonies at 125 μg/mL NAV concentration, probably due to the binding of NAV to amino groups on the bacterial surface, thereby reducing the bacterial number in the bacterial suspension after NAV treatment [43], while the NAV-NIR_(+)_ group showed a significant dose-dependent reduction in the number of bacterial colonies, with a bacterial inhibition rate as high as 99.6% at 125 μg/mL NAV concentration.

Moreover, the antibacterial activities of single NiO NPs, AuNPs@Van and AuNPs against MRSA were also evaluated by agar plate experiment. As shown in Fig. 4C, D and S2, incubation with NiO NPs plus exposure to NIR irradiation (NiO NPs-NIR_(+)_) showed a gradual decrease in the survival rate of MRSA, indicating the dose-dependent bactericidal activity of NiO NPs, with a survival rate of only 4.7% for MRSA after 125 μg/mL NiO NPs treatment. A possible explanation is that the photothermal and magnetic enrichment effects of NiO NPs played a synergistic antibacterial effect. Meanwhile, the number of colonies in the AuNPs-NIR_(+)_ (125 μg/mL) group showed a slight decrease, with an inhibition rate of 19.6% (Additional file 1: Fig. S2A and B). In Fig. 4E and F, the AuNPs@Van-NIR_(+)_ group was shown to have an antibacterial rate of 42.8% for MRSA at 125 μg/mL. Free spherical Au nanoparticles were reported to have optical resonance in the visible range, and when they are in the aggregation or orderly assembly state, coupled surface plasmons will appear between Au nanoparticles, enabling them to absorb NIR light and convert it into hyperthermia [53, 54]. In the present study, Van modified on the surface of AuNPs can identify Gram-positive bacteria, and during co-incubation of AuNPs and MRSA, Van-mediated AuNPs will self-aggregate on the surface of MRSA, absorbing light energy and turning it into heat to play the sterilization role under NIR irradiation. The above conclusion was confirmed by TEM analysis of the surface morphology of MRSA and *E. coli* treated with AuNPs@Van. In (Additional file 1: Fig. S3A and B), many AuNPs@Van particles were seen to be adsorbed on the surface of MRSA, but not on the surface of *E. coli.* Besides, in the MRSA and *E. coli* mixed bacteria suspension incubated with AuNPs@Van, only MRSA cells were surrounded by AuNPs@Van, while *E. coli* cells were almost free of AuNPs@Van (Additional file 1: Fig. S3C and D). The results showed that NAV could recognize MRSA, due to the mediating effect of Van modified on the AuNPs surface.

MIC was determined to confirm the in vitro antibacterial effect of NAV, NiO NPs and AuNPs@Van. As shown in (Additional file 1: Table S1), both NiO NPs-NIR_(+)_ and NAV-NIR_(+)_ could significantly inhibit MRSA at 125 μg/mL, and the MIC of both is lower than that of AuNPs@Van-NIR_(+)_, which further supports the results of the agar plate experiment.

The magnetic enrichment of bacteria by NiO NPs and NAV was also investigated. Briefly, 1.0 mL bacterial suspension (10^7^ CFU/mL) was added to sterile glass bottles, followed by adding to the bacterial suspension an equal volume of PBS, NiO NPs (125 μg/mL), and NAV (125 μg/mL), 5 min of magnetic absorption, and observing the liquid clarity in sterile glass bottles. In Additional file 1: Fig. S4A, the solution in the PBS group was seen to be in a typical turbid state, in contrast to a clear state for the solution in NiO NPs and NAV treatment groups, probably due to the magnetic properties of NiO and NAV in adsorbing bound bacteria. Additionally, we quantified the inhibition rates on MRSA for the supernatants of NiO NPs and NAV treatment groups to verify their antibacterial properties of magnetic adsorption. In Additional file 1: Fig. S4B and 4C, the magnetic adsorption inhibition rates on MRSA were shown to be 43.2% and 41.4% for NiO NPs and NAV, respectively. These results agreed well with the agar plate experimental observations, further indicating the successful construction of a synergistic antibacterial system due to the photothermal effect of Van-mediated self-aggregation of AuNPs, the photothermal effect of NiO NPs, and the magnetic enrichment effect.

The in vitro antibacterial activity of NAV was further evaluated using the live/dead double-stained fluorescent dye DAPI/PI. MRSA cells were stained with DAPI and PI, with DAPI (blue fluorescence) staining live cells, while PI (red) only staining cells with damaged walls. In Fig. 4G, the PBS-NIR_(−)_ and PBS-NIR_(+)_ groups showed strong green fluorescence for MRSA, with almost no red fluorescence being collected, suggesting the negligible effect of NIR laser treatment alone on the viability of bacteria. However, when the MRSA cells were treated with NAV-NIR_(−)_, an increasing number of red spots can be observed, and when NIR was introduced (NAV-NIR_(+)_), all the MRSA cells were stained red. The results indicated that PTT and magnetic enrichment synergistically endowed NAV with a strong bacteria-killing ability.

**Fig. 5:**
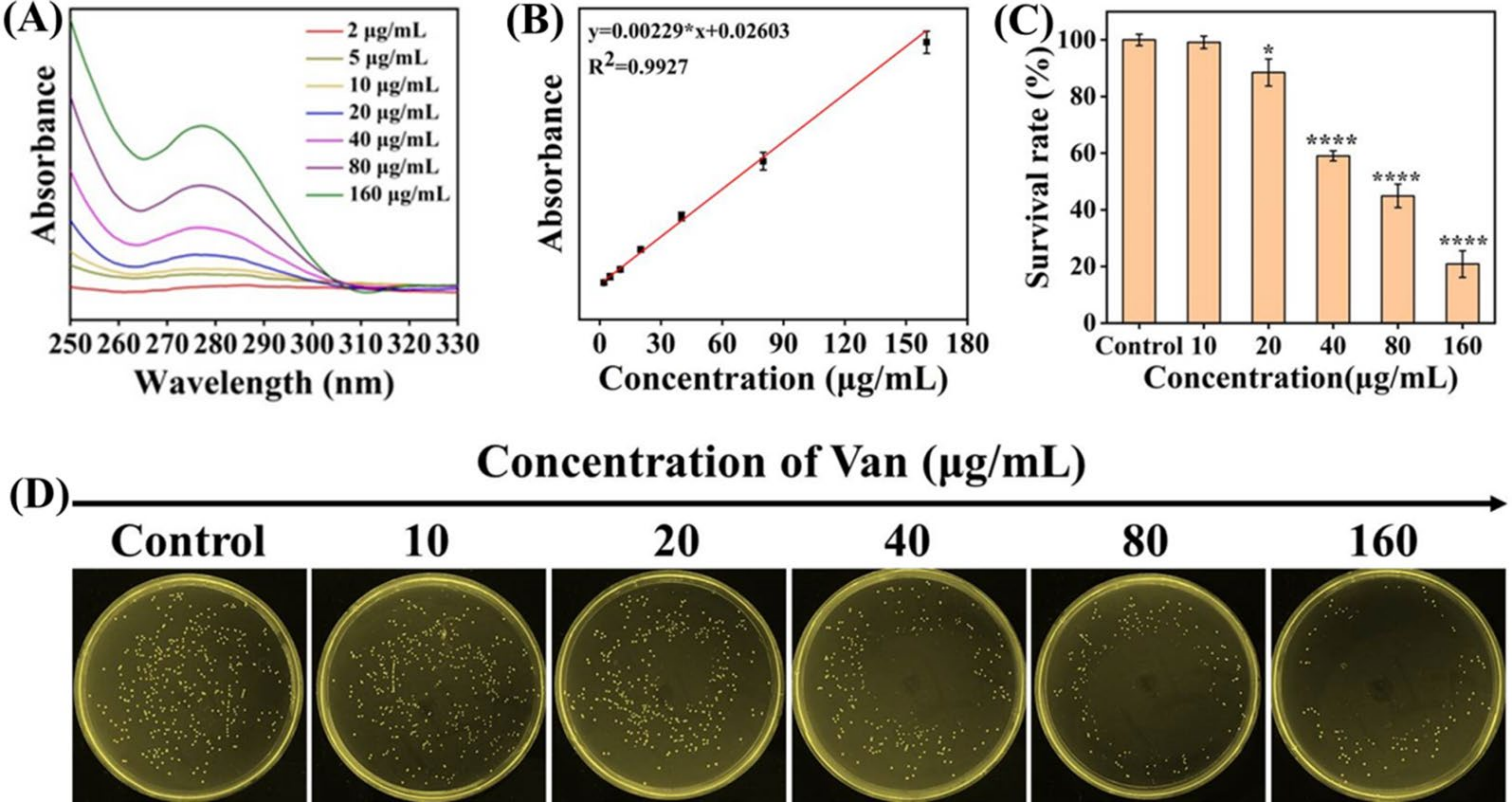
**A** UV–vis absorption spectra of Van at different concentrations (2–160 μg/mL). **B** The linear correlation between absorbance and Van concentrations. **C** The survival rate of MRSA exposed to different concentrations of Van. **D** The photograph of MRSA agar plate corresponding to **C** (see details in the text)

Van can be anchored on the bacterial surface by binding to the D-Ala-D-Ala part of the cell wall of Gram-positive bacteria, thereby actively targeting such bacteria [55, 56]. In this work, whether the Van modified with AuNPs surface plays a role in the antibacterial effect was verified by agar plate experiment. In Fig. 5A and B, the load of Van on the AuNPs surface was calculated to be 2.77 μg/mL. Then we measured the effect of different concentrations of Van on MRSA activity. In Fig. 5C and D, the MRSA activity was shown to be suppressed by Van in a dose-dependent manner. After exposure to 10 μg/mL Van, the MRSA survival rate was as high as 99.16%, proving the little effect of Van on bacterial growth at this concentration. The load of Van on AuNPs in our work was 2.77 μg/mL, far less than 10 μg/mL, so Van alone did not play a role in the strong antibacterial activity of NAV.

### Antibacterial mechanisms of NAV

**Fig. 6:**
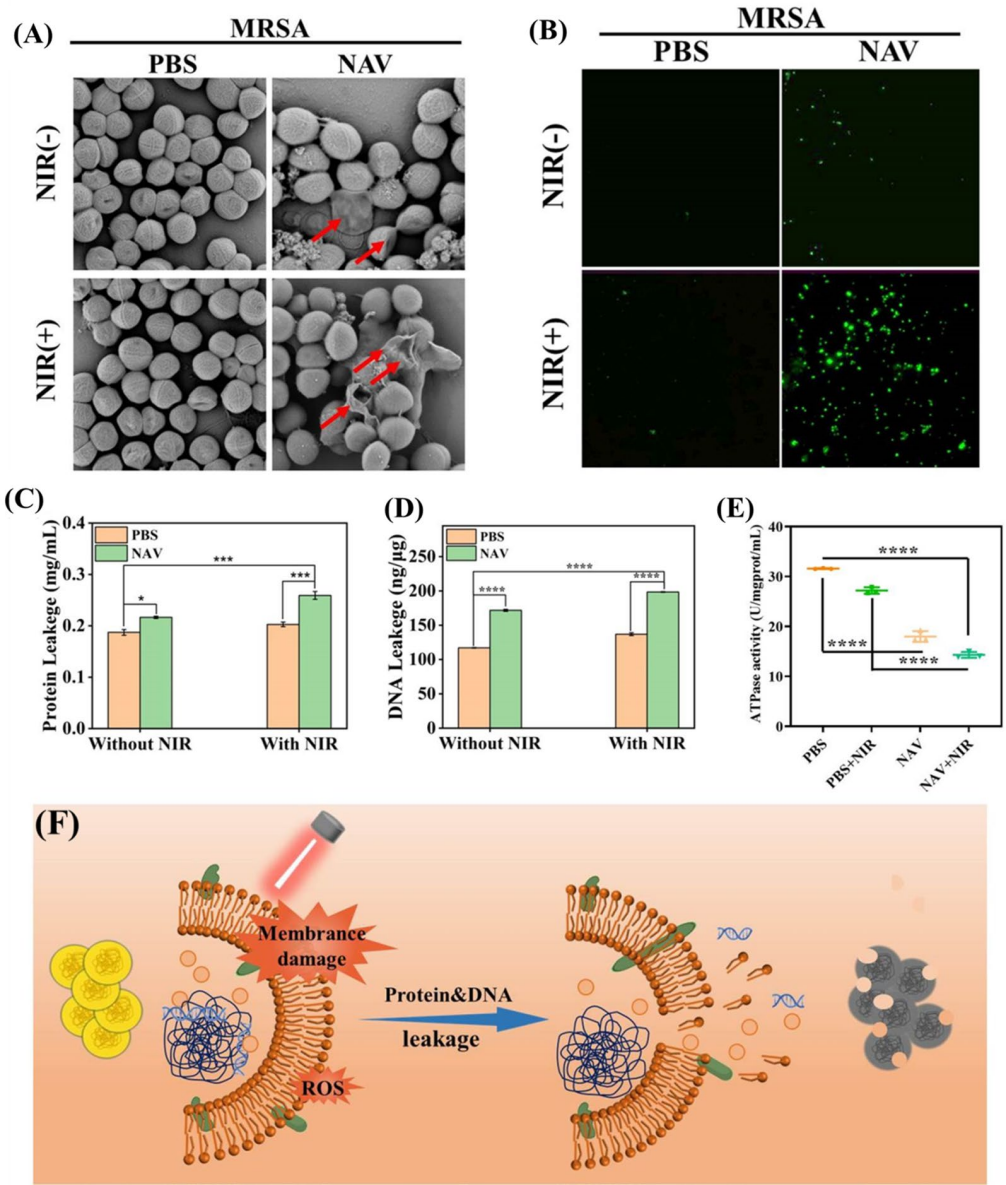
**A** Typical SEM images of MRSA treated with PBS (PBS-NIR_(−)_ and PBS-NIR_(+)_) and NAV (NAV-NIR_(−)_ and NAV-NIR_(+)_). **B** Intracellular ROS level of MRSA induced by NAV in the presence and absence of NIR laser irradiation. **C** Protein leakage of NAV-treated MRSA with or without NIR laser irradiation. **D** DNA leakage of NAV-treated MRSA with or without NIR laser irradiation. **E** The ATPase activity of MRSA after exposure to NAV with or without NIR laser irradiation. All the tests were performed at 125 μg/mL NAV concentration with or without 808 nm NIR laser irradiation at 1.8 W/cm^2^ for 10 min. **F** Schematic diagram for NAV antibacterial mechanism

The antibacterial mechanism of NAV was explored by SEM characterization of the bacterial cell morphology after different treatments. As shown in Fig. 6A, the morphology of MRSA was intact, with a smooth surface and a regular shape in the PBS group (either PBS-NIR_(–)_ or PBS-NIR_(+)_). Meanwhile, incubation of MRSA with NAV (NAV-NIR_(−)_) was shown to rupture the cell membranes of some bacteria, leading to membrane content leakage, but some bacteria still had good cell structure, suggesting the limited bactericidality of NAV. In contrast, the NAV-NIR_(+)_ treatment showed severe damage in the cell wall and membrane, leading to more obvious content leakage, implying stronger photothermal effect of NAV on MRSA cells under NIR irradiation. The destruction of cell membranes is usually considered a significant mark of disinfection [57, 58]. Additionally, we investigated the effects of AuNPs@Van, NiO NPs and NAV on the morphology of MRSA through TEM (Additional file 1: Fig. S5). MRSA maintained a complete shape and a regular smooth surface in the control group (PBS-NIR_(−)_ and PBS-NIR_(+)_), and incubation with AuNPs@Van or NiO NPs without NIR irradiation only made the cell membrane surface slightly twisted and wrinkled, demonstrating that AuNPs@Van or NiO NPs alone had little effect on the integrity of the bacterial cell membrane. In contrast, the AuNPs@Van-NIR_(+)_ and NiO NPs-NIR_(+)_ groups exhibited obvious cell deformation and content leakage, indicating that the photothermal effect strengthened the damage to bacteria. Compared with AuNPs@Van or NiO NPs treatment, exposure to NAV without NIR (NAV-NIR_(–)_) made the bacterial surface coarser and wrinkled, while exposure to NAV plus NIR (NAV-NIR_(+)_) caused the bacteria to completely lose their cell integrity, implying stronger antibacterial ability of the synergistic strategy.

Once the bacterial structure is destroyed, its intracellular components, including proteins, nucleic acid, will leak out. Figure 6C displays the protein leakage results after treating MRSA with 125 μg/mL NAV in the presence or absence of NIR laser irradiation. Compared with the PBS-NIR_(+)_ group, the NAV-NIR_(+)_ group had significantly (*p* < 0.001) higher protein leakage (0.21 mg/mL versus 0.26 mg/mL). As expected, DAN leakage obviously increased from 137.0 ng/μg (PBS-NIR_(+)_) to 198.6 ng/μg in response to the NAV-NIR_(+)_ treatment (Fig. 6D). Besides, the ATPase activity showed a 1.9-fold reduction in the NAV-NIR_(+)_ group relative to the PBS-NIR_(+)_ group (Fig. 6E). These results suggested that NIR-assisted NAV treatment could disrupt bacterial membranes, leading to the release of bacterial contents and ultimately the death of bacteria.

Moreover, the intracellular ROS level was also investigated, and disrupting the bacterial wall and cell membrane was not the only cause for bacterial death. As depicted in Fig. 6B, the intracellular ROS increased significantly after incubation with 125 μg/mL NAV, and the green fluorescence signal further increased after treatment of NAV plus 808 nm NIR laser, implying that NAV-NIR_(+)_ treatment could stimulate in situ ROS production in MRSA. This observation was consistent with the aforementioned agar plate and MIC results. Based on the above results and discussion, the antibacterial mechanism of NAV against MRSA is visually demonstrated in Fig. 6F. Endogenously generated ROS plays an important role in signal transduction. Specifically, when the generated ROS exceeds the cell clearance capacity, a chain reaction will be triggered: ROS will oxidize the intracellular proteins through the bacterial cell wall and destroy the bacterial homeostasis, and the hyperthermia generated by NAV under 808 nm laser irradiation will reduce the protein activity and ATPase activity, making joint contributions to bacterial death [59].

### In vivo antibacterial activity of NAV and skin wound healing

Inspired by the excellent antibacterial efficacy of NAV in vitro experiments, we further investigated the application prospects of NAV in animal wound disinfection and tissue healing. Before treatment, whether NAV is safe was tested by injecting NAV (200 μL, 125 μg/mL) into mice and assessing its acute toxicity. The results of serum biochemistry tests showed no statistically significant difference in the values of aspartate aminotransferase (AST) (Additional file 1: Fig. S6A), alkaline phosphatase (ALP) (Additional file 1: Fig. S6B), albumin (ALB) (Additional file 1: Fig. S6C) and Creatinine (Cr) (Additional file 1: Fig. S6D) in the control and NAV treatment groups, indicating no apparent toxicity of NAV at a given dose. Additionally, H&E staining showed no statistically significant differences in the internal organs of the mice (heart, liver, spleen, lung, kidney) (Additional file 1: Fig. S6E). The above results indicated that NAV (200 μL, 125 μg/mL) can be safely used in animal experiments under our experimental conditions.

**Fig. 7:**
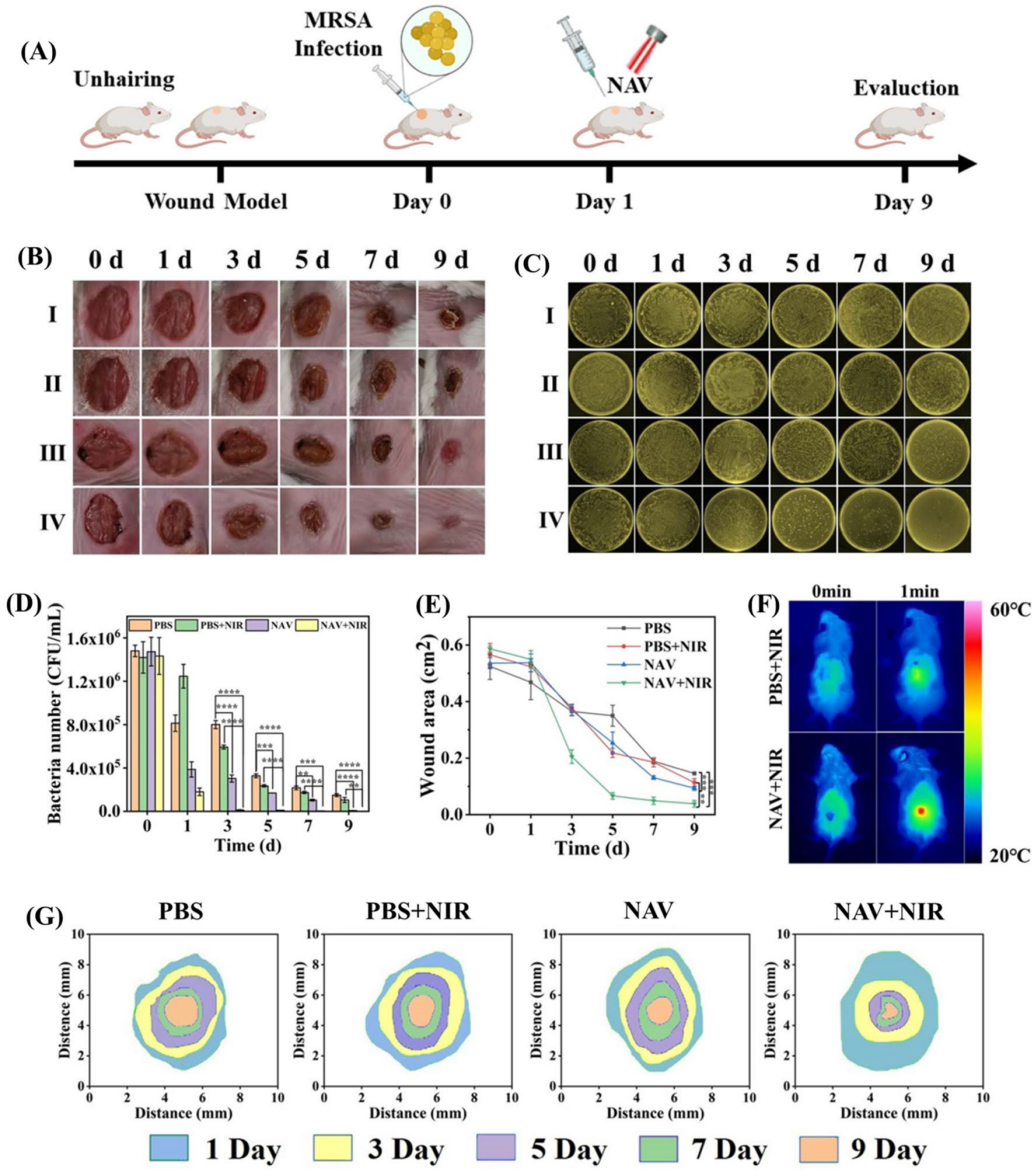
**A** The schematic diagram of wound model and treatment strategy of MRSA infection. **B** Photographs of MRSA-infected skins treated with I (PBS-NIR_(−)_), II (PBS-NIR_(+)_), III (NAV-NIR_(−)_) and IV (NAV-NIR_(+)_) after 0, 1, 3, 5, 7 and 9 days of therapy. **C** Corresponding agar plate experiment pictures of MRSA-infected skin in different treatment groups after 0, 1, 3, 5, 7 and 9 days of treatment. **D** The inhibition rate on bacterial growth for different treatment groups at different time intervals corresponding to **C**. **E** Changes in the wound area of infected mice from day 0 to 9 during treatment. **F** Infrared thermal images of mice treated with NAV before and after 808 nm NIR (1.8 W/cm^2^) irradiation for 1 min. **G** The dynamic process of wound healing in each treatment group from day 1 to 9

Then the BALB/c mice infected with MRSA were used as a full-thickness skin defect repair model (Fig. 7A). The infected mice were randomly divided into 4 groups: (I (PBS-NIR_(-)_), (II (PBS-NIR_(+)_), (III (NAV-NIR_(−)_), and (IV (NAV-NIR_(+)_). In Fig. 7F, the temperature of the mouse wound area was seen to increase to 55.3 °C in the NAV-NIR_(+)_ group after 1 min of irradiation, intuitively proving that NAV can be used as a photothermal antibacterial agent. Meanwhile, no significant temperature changes were detected in the PBS-NIR_(+)_ group. This result was consistent with the in vitro photothermal experiment. A possible explanation for the temperature changes is that the surface plasmon resonance effect activated the aggregation of AuNPs mediated by Van and NiO NPs synergistically [60]. In this study, the photographs of the mice infected parts were taken on day 0, 1, 3, 5, 7 and 9 of treatment (Fig. 7B). On day 0, redness and swelling could be seen in the infected area of all groups. With the passage of treatment time, the scars of all groups gradually darkened in color and gradually reduced in size, demonstrating that the epidermal bacterial infection was suppressed and the wounds were gradually healed. The NAV-NIR_(−)_ and NAV-NIR_(+)_ groups showed significantly better scar rates than the other two groups. After 9 days of treatment, the wound in the NAV-NIR_(−)_ group had scabs and became significantly smaller, but the infected site was still swollen, indicating that NAV has a certain antibacterial ability, but it is not enough to cure MRSA skin infections. In contrast, the scabs in the NAV-NIR_(+)_ group were healed and fell off, with the wounds being basically healed, further proving that the antibacterial ability of NAV was enhanced under NIR irradiation. The antibacterial performance of each treatment group was further quantified by monitoring the number of colonies formed at the infected site during the treatment. In Fig. 7C, no obvious difference could be presented in the number of colonies between PBS-NIR_(−)_ and PBS-NIR_(+)_ groups, indicating that NIR does not affect the bacterial growth. However, with the extension of treatment time, the number of colonies gradually decreased in the NAV-NIR_(−)_ and NAV-NIR_(+)_ groups. After 9 days of treatment, the NAV-NIR_(−)_ group and NAV-NIR_(+)_ group showed a decrease of 95.5% and 99.7% in the number of colonies as compared to the PBS-NIR_(−)_ group (Fig. 7D). Furthermore, on day 9, the wound area was 0.146 ± 0.004 cm^2^ in the PBS-NIR_(-)_ group, but 0.093 ± 0.007 cm^2^ and 0.038 ± 0.011 cm^2^ in the NAV-NIR_(−)_ and NAV-NIR_(+)_ groups, respectively, corresponding to the healing rate of 82.6% and 93.5% (Fig. 7E and Additional file 1: Table S2), with Fig. 7G showing the real-time tracking dynamic diagram for the infected area of each treatment group. The above results indicated that the synergistic treatment based on the photothermal effect of Van-mediated AuNPs aggregation and the photothermal effect of NiO NPs can effectively combat MRSA skin infections.

**Fig. 8:**
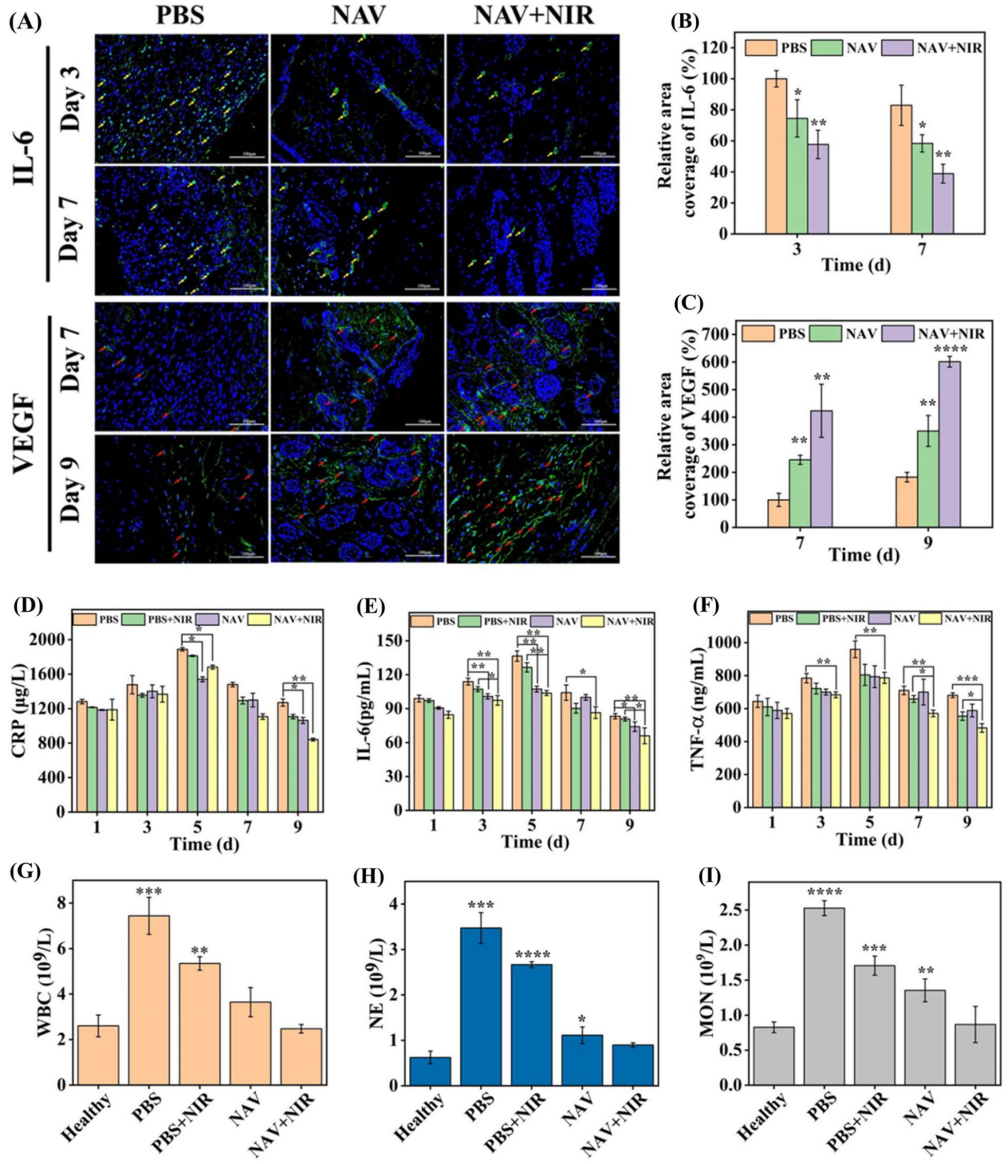
**A** Immunofluorescence staining of regenerative wound tissues with IL-6 on day 3 and 7 and VEGF on day 7 and 9, with the yellow arrow for the expression of IL-6 and the red arrow for the expression of VEGF. The corresponding quantitative data of IL-6 **B** and VEGF **C** expression. The expression levels of C-reactive protein (CRP) **D**, IL-6 **E** and TNF-α **F** in MRSA-infected mice from day 1–9 in different treatment groups. Changes of white blood cell (WBC) **G**, neutrophil (NE) **H** and monocyte (MON) **I** levels in different treatment groups

Studies have shown that the cell activities regulated by cytokines affect the wound healing process [61]. Interleukin 6 (IL-6) and vascular endothelial growth factor (VEGF) are usually used as important indicators in mouse skin defect models to evaluate the effect of a dressing on wound healing. IL-6, a pro-inflammatory cytokine, is closely related to the degree of inflammation in the early stage of wound healing (day 3 and 7). VEGF, an important angiogenic factor, can reflect the angiogenesis in the late stage of wound healing (day 7 and 9). Quantification of immunofluorescence stained wound area indicated that IL-6 expression gradually decreased with the passage of treatment time (Fig. 8A and B). Compared with the PBS group, there was a significant difference between NAV-NIR_(−)_ and NAV-NIR_(+)_ groups in IL-6 expression on day 3 and 7, with a lower IL-6 expression in the NAV-NIR_(+)_ group than in the NAV-NIR_(−)_ group, indicating that the synergistic effect between the photothermal properties of NAV and the inherent bactericidal activity of NiO NPs enhanced the bactericidal effect. Figure 8A and C show the immunofluorescence staining and expression quantification results of VEGF. VEGF was seen to increase gradually on day 7 and 9 of treatment, and the NAV-NIR_(+)_ group showed the highest VEGF expression level in the three test groups.

Additionally, we quantified the changes in serum C-reactive protein (CRP), IL-6 and TNF-α levels in different treatment groups at different post-infection time points as a substitute index for further evaluation of mice recovery. CRP is a sensitive indicator of bacterial infection. In Fig. 8D, CRP was seen to reach the highest level in each group on day 5 of MRSA infection, followed by a gradual decrease. The CRP content was 1270.7 ± 41.8 μg/L for the PBS-NIR_(−)_ group, but was significantly reduced to 841.1 ± 16.1 μg/L in the NAV-NIR_(+)_ group on day 9. As expected, during the wound healing process, the expression of IL-6 (Fig. 8E) and TNF-α (Fig. 8F) reached the maximum in each treatment group on day 5, and with the extension of treatment time, the expression levels of TNF-α and IL-6 decreased significantly in the NAV-NIR_(+)_ group relative to the PBS-NIR_(−)_ group, namely 65.9 ± 7.0 pg/mL vs 83.4 ± 2.4 pg/mL (PBS-NIR_(−)_ group) and 482.5 ± 26.0 ng/mL vs 680.8 ± 14.5 ng/mL (PBS-NIR_(−)_ group) on day 9, respectively. These results further demonstrated that NAV plus NIR treatment could accelerate the healing of MRSA-infected wounds in mice by down-regulating the expression of inflammatory cytokines.

When the mice are infected by bacteria, the body itself initiates an immune response and recruits a large number of immune cells to fight the infection. As presented in Fig. 8G, H and I white blood cells (WBCs), neutrophils (NEs) and monocytes (MONs) were still at a relatively high level in the PBS-NIR_(−)_ group, indicating that the mice were still in the state of infection response. However, the immune cells were reduced to almost the same level as in healthy mice in the NAV-NIR_(+)_ group, implying that the combination of NAV and NIR therapy could effectively eliminate infection and inflammation in mice.

**Fig. 9:**
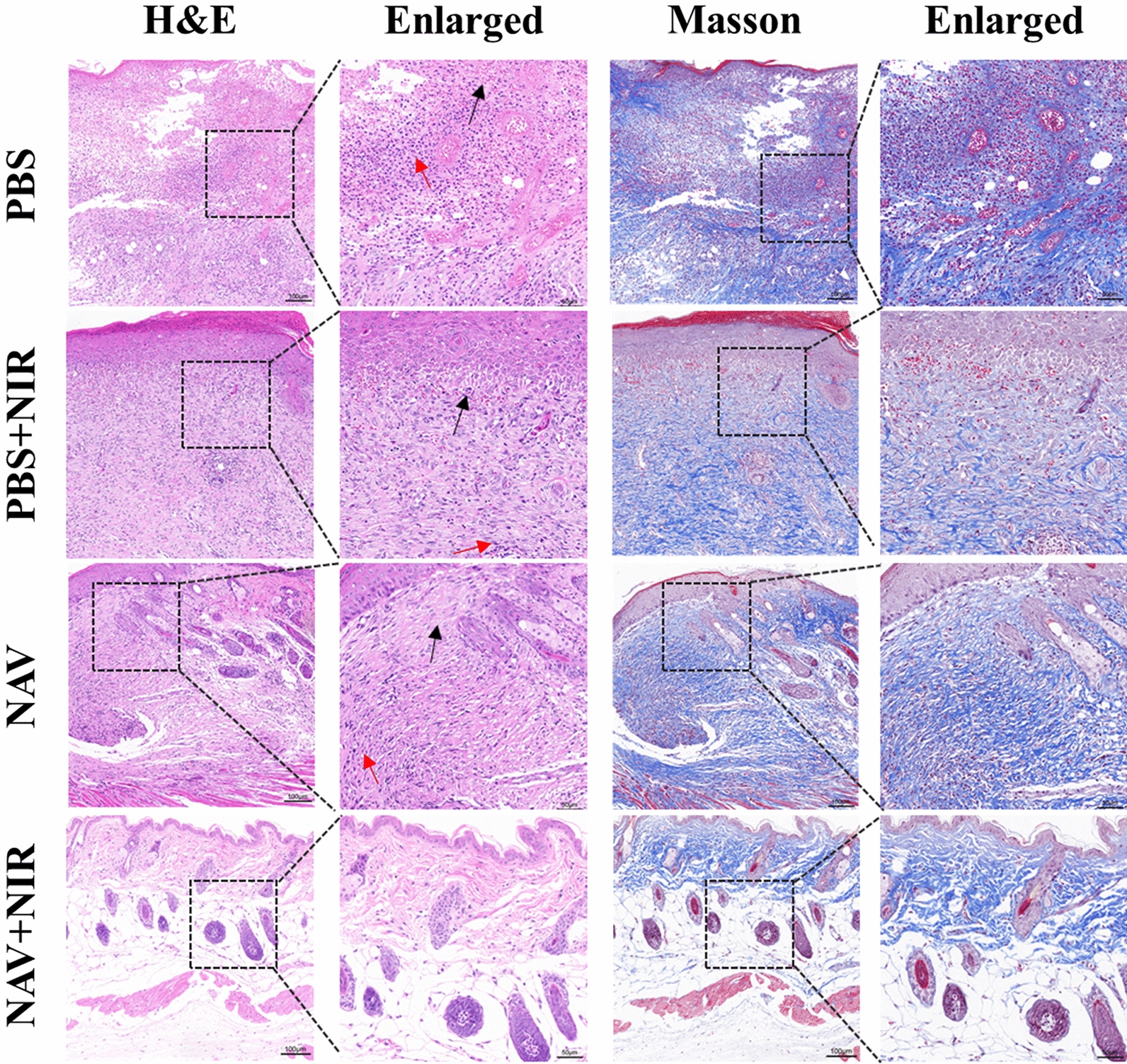
The histological observation of skin wound in different treatment groups at day 9 of treatment based on H&E staining and Masson staining analysis (see details in the text)

The above results were further verified by analyzing the histological changes of skin tissues. Hematoxylin & eosin (H&E) and Masson’s trichrome (MT) staining showed that in the PBS-NIR_(−)_ group, the skin tissue structure was moderately abnormal, with some areas of epidermis being necrotic and exfoliated and collagen fibers in the dermis being reduced in number and disordered with bleeding (shown by black arrows), couple with massive infiltration of inflammatory cells, mainly neutrophils (indicated by red arrows). In the PBS-NIR_(+)_ group, the arrangement of collagen fibers was disordered in some areas of the epidermis and dermis (shown by black arrows), and inflammatory cell infiltration could be observed (indicated by red arrows). Compared with the PBS-NIR_(−)_ group, the skin tissue structure of the NAV-NIR_(–)_ group was significantly repaired after 9 days of treatment, and the collagen fibers were significantly increased (shown by black arrows), with a small amount of observable inflammatory cell infiltration (indicated by red arrows). In contrast, the skin tissue structure of the NAV-NIR_(+)_ group was basically normal, the collagen fibers were arranged neatly and tightly, and there was no obvious inflammatory cell infiltration (Fig. 9). Collagen fibers, the main component in the dermis, are usually deposited in the marginal space of the wound during wound healing. Therefore, all the data demonstrated that the combination of NIR and NAV can eliminate bacteria and accelerate the wound healing process of MRSA-infected mice, suggesting the potential use of NAV as an effective therapeutic agent for treating infected wounds in vivo*.*

In this study, we further assessed the in vivo wound healing ability of PBS-NIR_(–),_ AuNPs-NIR_(−)_, AuNPs-NIR_(+)_, AuNPs@Van-NIR_(−)_, AuNPs@Van-NIR_(+)_, NiO NPs-NIR_(−)_, and NiO NPs-NIR_(+)_) treatment groups. Additional file 1: Fig. S7A displays a representative photograph of wound closure for different treatment groups collected every two days during the 9 days of experiment. In Additional file 1: Fig. S7C, it was shown that compared with the control group (PBS-NIR_(–)_), the wound was significantly smaller in the AuNPs@Van-NIR_(+)_, NiO NPs-NIR_(–)_, and NiO NPs-NIR_(+)_ groups, especially the NiO NPs-NIR_(+)_ group, where the wound area recovered from 0.51 ± 0.01 cm^2^ to 0.04 ± 0.003 cm^2^, probably due to the magnetic thermal effect of NiO NPs. For quantifying the antibacterial performance of each treatment group, the number of colonies formed at the infection site during treatment was calculated for each treatment group (Additional file 1: Fig. S7B and D). After 9 days of treatment, AuNPs@Van-NIR_(+)_, NiO NPs-NIR_(–)_, NiO NPs-NIR_(+)_ groups showed obvious inhibitory effects, with an inhibition rate of 41.7%, 89.6% and 99.0%, respectively (Additional file 1: Fig. S7D). Compared with the PBS group, AuNPs@Van-NIR_(+)_, NiO NPs-NIR_(–)_ and NiO NPs-NIR_(+)_ groups showed significant reduction in the level of neutrophils (NEs) (Additional file 1: Fig. S7E), monocytes (MONs) (Additional file 1: Fig. S7F), and white blood cells (WBC) (Additional file 1: Fig. S7G), suggesting that the three treatments could effectively suppress bacterial infection. Additionally, all the treatment groups showed no significant changes in red blood cells (RBC) (Additional file 1: Fig. S7I), mean corpuscular volume (MCV) (Additional file 1: Fig. S7J), mean corpuscular volume (MCH) (Additional file 1: Fig. S7K), hematocrit (HCT) (Additional file 1: Fig. S7L) index and body weight (Additional file 1: Fig. S7H) in each treatment group, further indicating the materials have excellent blood biocompatibility.

### Biosafety evaluation

**Fig. 10:**
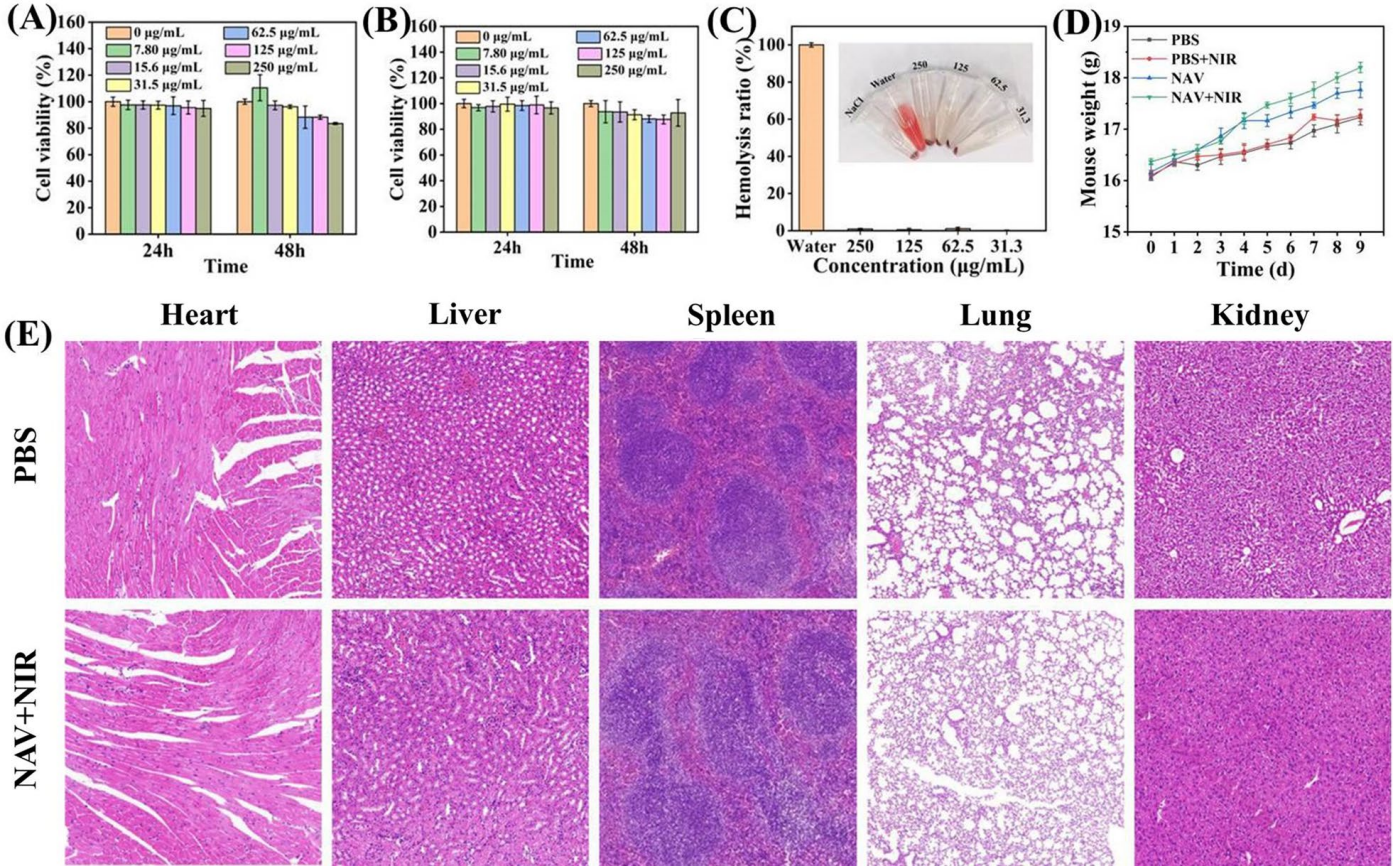
The viability of **A** Vero cells and **B** NIH 3T3 cells after 24 and 48 h of incubation with different concentrations of NAV. **C** The hemolysis ratio of water and different concentrations of NAV (31.3–250 μg/mL) with corresponding photos. **D** Changes in the body weight of MRSA-infected mice from day 0 to 9 during treatment. **E** **H**&**E** staining analysis of the tissue sections of major organs (heart, liver, spleen, lung and kidney) after 9 days of treatment

In addition to antibacterial effects, biological safety is another criterion for investigating the potential of an antibacterial agent in clinical applications. For biosafety test, the cytotoxicity of different concentrations of NAV on Vero cells and NIH 3T3 cells were first determined by the MTT method. In Fig. 10A and B, the cells co-cultured for 48 h with NAV at 250 μg/mL concentration showed a cell viability of > 83%, indicating that NAV has good cell compatibility at the experimental dose [62]. Additionally, different concentrations of NAV were incubated with mouse red blood cells to further evaluate the hemocompatibility of NAV by in vitro hemolysis test. As shown in Fig. 10C, the supernatant color was dark red for the positive control group (water), but clear for the negative control group (NaCl) and NAV groups of various concentrations (250, 125, 62.5, and 31.3 μg/mL). The corresponding quantitative data showed that with the increase of NAV concentration, the hemolysis rate of red blood cells increased slightly (0.04%-0.87%), with a hemolysis rate of 0.87% for NAV at the concentration as high as 250 μg/mL, suggesting that NAV has good blood compatibility [63].

The in vivo biological safety of NAV was further discussed. As suggested in Fig. 10D, during the 9 days of treatment, the four groups of mice showed no significant change in the body weight, indicating that NAV had no evident biological toxicity. Meanwhile, whole blood and serum in the treatment and blank groups showed no significant changes in the hematological parameters, including alanine transaminase (ALT) (Additional file 1: Fig. S8A), aspartate transaminase (AST) (Additional file 1: Fig. S8B), creatinine (Additional file 1: Fig. S8C), alkaline phosphatase (ALP) (Additional file 1: Fig. S8D), albumin (ALB) (Additional file 1: Fig. S8E), red blood cells (RBC) (Additional file 1: Fig. S9A), mean corpuscular volume (MCH) (Additional file 1: Fig. S9B), hemoglobin (HGB) (Additional file 1: Fig. S9C), hematocrit (HCT) (Additional file 1: Fig. S9D) and mean corpuscular volume (MCV) (Additional file 1: Fig. S9E), further indicating that NAV had good blood compatibility. Moreover, H&E staining analysis of the heart, liver, spleen, lung and kidney showed no significant differences among the 4 groups in these tissues, verifying that NAV treatment does not cause obvious inflammation or pathological abnormality in tissues (Fig. 10E). Furthermore, free Au^3+^ and Ni^2+^ in lung and liver were quantified by ICP-MS. The free Au^3+^ was 0.019 and 0.003 μg/mL and the free Ni^2+^ was 1.952 and 0.038 μg/mL for the lung and liver, respectively. Meanwhile, the cytotoxicity of free Au^3+^ and Ni^2+^ was also investigated, and as shown in Additional file 1: Fig. S10, no cytotoxicity was detected after 48 h of co-incubation of Vero cells and NIH/3T3 cells with different concentrations of Au^3+^ and Ni^2+^. These results proved that NAV has good biological safety in vitro and in vivo.

## Conclusion

In this work, NiO NPs@AuNPs@Van (NAV), a highly effective antibacterial nanocomposite targeting the recognition of MRSA, was successfully constructed by electrostatic self-assembly of magnetic NiO NPs and Van-modified AuNPs. The antibacterial mechanisms of NAV can be summarized as follows: (1) in the presence of MRSA and under near-infrared light irradiation, Van-mediated spherical AuNPs self-aggregated on the surface of MRSA will activate plasmon resonance between particles to generate heat; (2) NiO NPs, an outstanding NIR-assisted photothermal agent, can enhance the photothermal effect synergistically with aggregated AuNPs; (3) the excellent magnetic properties of NAV can further enhance the photothermal effect through magnetic enrichment, facilitating targeted killing of MRSA at a lower dose. Therefore, the hyperthermia caused the damage and deformation of cell membrane and the leakage of contents, leading to the ultimate death of bacteria. In vitro and in vivo test results indicated that the NAV had prominent bactericidal properties and excellent biosafety and could promote wound healing. Considering these advantages, NAV may provide a novel perspective for treating both medical device-associated and wound infections.

## Supplementary Information

**Additional file 1: Figure S1.** EDS analysis of NAV.** Table S1.** MIC of NiO NPs, AuNPs@Van and NAV against MRSA under 808 nm NIR irradiation. **Figure S2.** (A) The survival rate of MRSA after treatment with different concentrations (7.80, 15.6, 31.3, 62.5, 125 and 250 μg/mL) of AuNPs (AuNPs-NIR_(-)_ and AuNPs-NIR_(+)_). (B) Photographs of colony formation of MRSA corresponding to (A). **Figure S3. **TEM images of (A) MRSA and (B) *E. coli* treated with AuNPs@Van at 125 μg/mL. (C and D) TEM images of MRSA and* E. coli *mixed bacteria suspension treated with AuNPs@Van at 125 μg/mL. **Figure S4.** Antibacterial activity and binding ability of NiO NPs and NAV. (A) Binding of MRSA by NiO NPs and NAV. (B) The survival rate of MRSA after treatment with NiO NPs and NAV. (C) Photographs of colony formation of MRSA corresponding to (B). **Figure S5.** Morphologies of MRSA treated separately by PBS-NIR_(-)_, PBS-NIR_(+)_, AuNPs@Van-NIR_(-)_, NiO NPs-NIR_(-)_, NiO NPs -NIR_(+)_, NAV-NIR_(-)_ and NAV-NIR_(+)_ at 125 μg/mL with (NIR_(+)_) or without (NIR_(-)_) 808 nm NIR irradiation for 10 min. **Figure S6. **The toxicity assay of NAV *in vivo*. Changes of major serum biochemistry indicators of aspartate transaminase (AST) (A), alkaline phosphatase (ALP) (B), albumin (ALB) (C) and creatinine (D) in normal mice after treated by PBS and NAV. (E) The H&E staining of internal organs (heart, liver, spleen, lung and kidney) after treated by PBS and NAV. **Table S2. **The wound healing rate of different treatment groups. **Figure S7. **(A) Photographs of MRSA-infected skins treated with I (PBS-NIR_(-)_), II (AuNPs-NIR_(-)_), III (AuNPs-NIR_(+)_), IV (AuNPs@Van-NIR_(-)_), V(AuNPs@Van-NIR_(+)_), VI (NiO NPs-NIR_(-)_), VII (NiO NPs-NIR_(+)_) after 0, 1, 3, 5, 7 and 9 days of therapy. (B) Corresponding agar plate experiment pictures of MRSA-infected skin in different treatment groups after 0, 1, 3, 5, 7 and 9 days of treatment. (C) Changes in the wound area of infected mice from day 0 to 9 during treatment. (D) The inhibition rate on bacterial growth for different treatment groups at different time intervals corresponding to (B). Changes of neutrophil (NE) (E), monocyte (MON) (F) and white blood cell (WBC) (G) levels in different treatment groups. (H) Changes in the body weight of MRSA-infected mice from day 0 to 9 during treatment. Changes of MRSA-infected mice in (I) red blood cells (RBC), (J) mean corpuscular volume (MCV), (K) mean corpuscular volume (MCH) and (L) hematocrit (HCT) after different treatments. **Figure S8. **Changes of major serum biochemistry indicators of alanine transaminase (ALT) (A), aspartate transaminase (AST) (B), creatinine (C), alkaline phosphatase (ALP) (D), albumin (ALB) (E) in MRSA-infected mice after 9 days of treatment. **Figure S9. **Changes of MRSA-infected mice in (A) red blood cells (RBC), (B) mean corpuscular volume (MCH), (C) hemoglobin (HGB), (D) hematocrit (HCT) and (E) mean corpuscular volume (MCV) after different treatments. **Figure S10. **The viability of NIH 3T3 cells and Vero cells after 48 h of incubation with different concentrations of (A) Au^3+^ and (B) Ni^2+^.

## Abbreviations

NAV: NiO NPs@AuNPs@Van
MRSA: Methicillin-resistant *Staphylococcus aureus*
PTT: Photothermal therapy
EDC: 1-(3-Dimethylaminopropyl)-3-ethylcarbodiimide hydrochloride
NHS: N-Hydroxysuccinimide
DEG: Diethylene glycol
MIC: Minimum inhibitory concentration
ROS: Reactive oxygen species
RBC: Red blood cells
TEM: Transmission electron microscope
SEM: Scanning electron microscope
EDS: Energy dispersive spectroscopy
XRD: X-ray diffraction
XPS: X-ray photoelectron spectroscopy
FTIR: Fourier-transform infrared spectroscopy
ATP: Adenosine triphosphate
VEGF: Vascular endothelial growth factor
IL-6: Interleukin 6
CRP: C-reactive protein
WBC: White blood cell
NE: Neutrophil
MON: Monocyte
H&E: Hematoxylin & eosin
MT: Masson’s trichrome
MCH: Mean corpuscular volume
HGB: Hemoglobin
HCT: Hematocrit
MCV: Mean corpuscular volume
