# Supplementary material for: Van-mediated self-aggregating photothermal agents combined with multifunctional magnetic nickel oxide nanoparticles for precise elimination of bacterial infections
Source: J Nanobiotechnology. 2022 Jul 14;20:325. doi: 10.1186/s12951-022-01535-1 (PMC9281033; doi:10.1186/s12951-022-01535-1)
Supplement: Supplementary file 1 — Additional file 1: Figure S1. EDS analysis of NAV. Table S1. MIC of NiO NPs, AuNPs@Van and NAV against MRSA under 808 nm NIR irradiation. Figure S2. (A) The survival rate of MRSA after treatment with different concentrations (7.80, 15.6, 31.3, 62.5, 125 and 250 μg/mL) of AuNPs (AuNPs-NIR(-) and AuNPs-NIR(+)). (B) Photographs of colony formation of MRSA corresponding to (A). Figure S3. TEM images of (A) MRSA and (B) E. coli treated with AuNPs@Van at 125 μg/mL. (C and D) TEM images of MRSA and E. coli mixed bacteria suspension treated with AuNPs@Van at 125 μg/mL. Figure S4. Antibacterial activity and binding ability of NiO NPs and NAV. (A) Binding of MRSA by NiO NPs and NAV. (B) The survival rate of MRSA after treatment with NiO NPs and NAV. (C) Photographs of colony formation of MRSA corresponding to (B). Figure S5. Morphologies of MRSA treated separately by PBS-NIR(-), PBS-NIR(+), AuNPs@Van-NIR(-), NiO NPs-NIR(-), NiO NPs -NIR(+), NAV-NIR(-) and NAV-NIR(+) at 125 μg/mL with (NIR(+)) or without (NIR(-)) 808 nm NIR irradiation for 10 min. Figure S6. The toxicity assay of NAV in vivo. Changes of major serum biochemistry indicators of aspartate transaminase (AST) (A), alkaline phosphatase (ALP) (B), albumin (ALB) (C) and creatinine (D) in normal mice after treated by PBS and NAV. (E) The H&E staining of internal organs (heart, liver, spleen, lung and kidney) after treated by PBS and NAV. Table S2. The wound healing rate of different treatment groups. Figure S7. (A) Photographs of MRSA-infected skins treated with I (PBS-NIR(-)), II (AuNPs-NIR(-)), III (AuNPs-NIR(+)), IV (AuNPs@Van-NIR(-)), V(AuNPs@Van-NIR(+)), VI (NiO NPs-NIR(-)), VII (NiO NPs-NIR(+)) after 0, 1, 3, 5, 7 and 9 days of therapy. (B) Corresponding agar plate experiment pictures of MRSA-infected skin in different treatment groups after 0, 1, 3, 5, 7 and 9 days of treatment. (C) Changes in the wound area of infected mice from day 0 to 9 during treatment. (D) The inhibition rate on bacterial growth for differe [file 12951_2022_1535_MOESM1_ESM.docx]

Van-mediated self-aggregating photothermal agents combined with multifunctional magnetic nickel oxide nanoparticles for precise elimination of bacterial infections

Ting Du^^[[1]](#footnote-1)^^†, Jiangli Cao^1^†, Zehui Xiao^1^, Jiaqi Liu^1^, Lifei Wei^1^, Chunqiao Li^1^, Jingbo Jiao^1^, Zhiyong Song^3^, Jifeng Liu^1^, Xinjun Du^1,^*, Shuo Wang^2,^*

State Key Laboratory of Food Nutrition and Safety, Key Laboratory of Food Nutrition and Safety, Ministry of Education, College of Food Science and Engineering, College of Food Science and Engineering, Tianjin University of Science and Technology, Tianjin 300457, PR China.

^2^Tianjin Key Laboratory of Food Science and Health, School of Medicine, Nankai University, Tianjin 300071, PR China.

^3^College of Sicence, Huazhong Agricultural University, Wuhan 430070, PR China.

**Results**


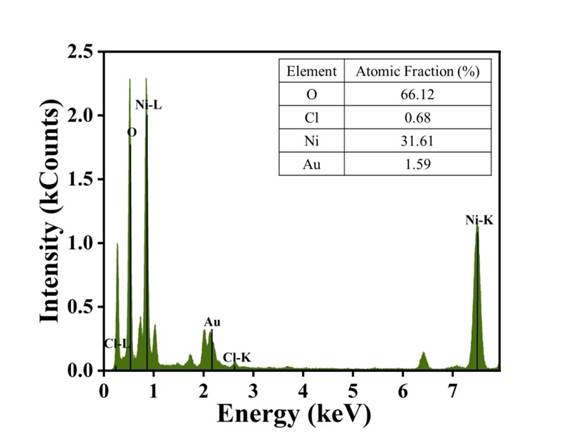


**Fig. S1** EDS analysis of NAV.

**Table S1.** MIC of NiO NPs, AuNPs@Van and NAV against MRSA under 808 nm NIR irradiation.

| **MIC** | NiO NPs+NIR | AuNPs@Van+NIR | NAV+NIR |
| --- | --- | --- | --- |
| MRSA | 125 μg/mL | ＞250 μg/mL | 125 μg/mL |


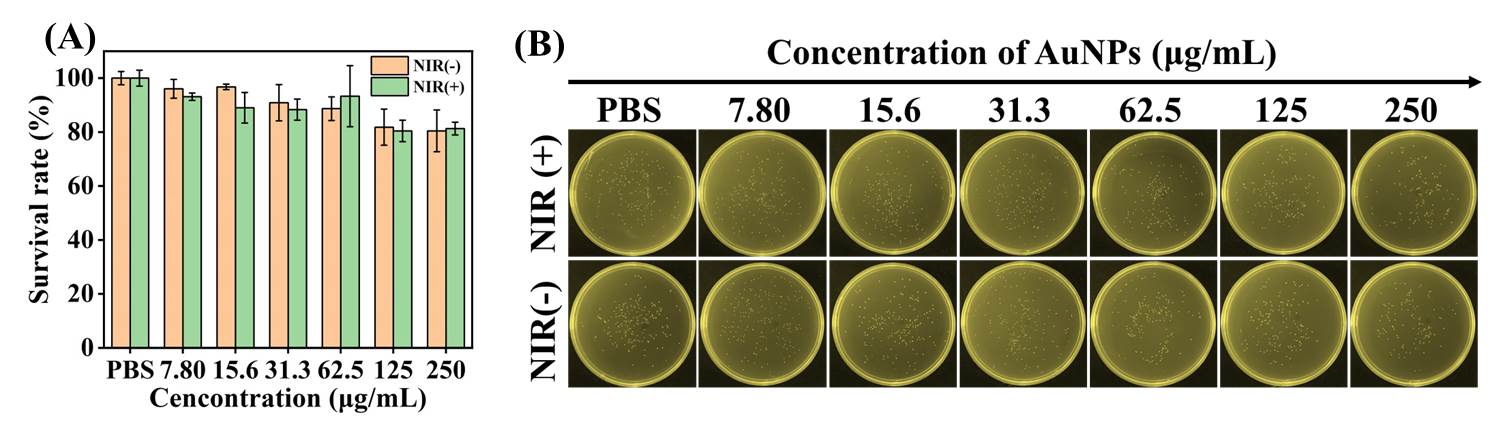


**Fig. S2** (A) The survival rate of MRSA after treatment with different concentrations (7.80, 15.6, 31.3, 62.5, 125 and 250 μg/mL) of AuNPs (AuNPs-NIR_(-)_ and AuNPs-NIR_(+)_). (B) Photographs of colony formation of MRSA corresponding to (A).


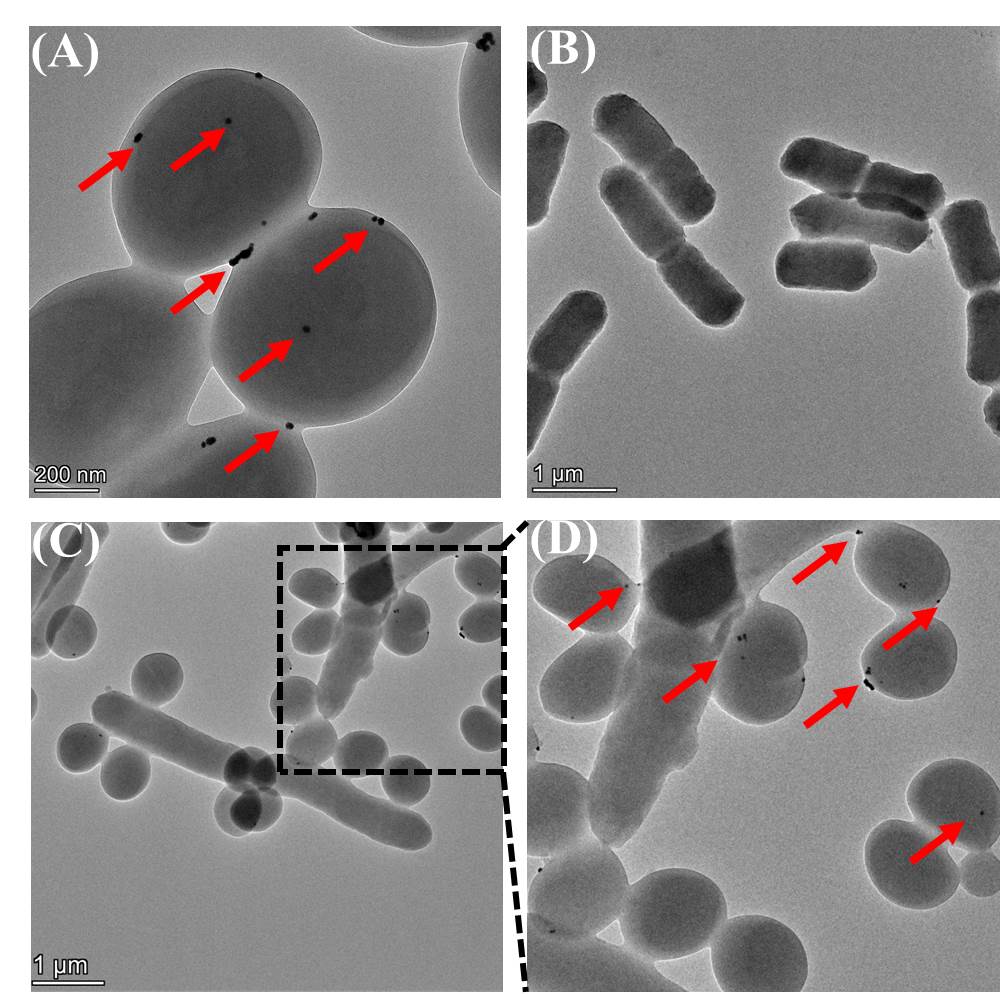


**Fig. S3** TEM images of (A) MRSA and (B) *E. coli* treated with AuNPs@Van at 125 μg/mL. (C and D) TEM images of MRSA and *E. coli* mixed bacteria suspension treated with AuNPs@Van at 125 μg/mL.


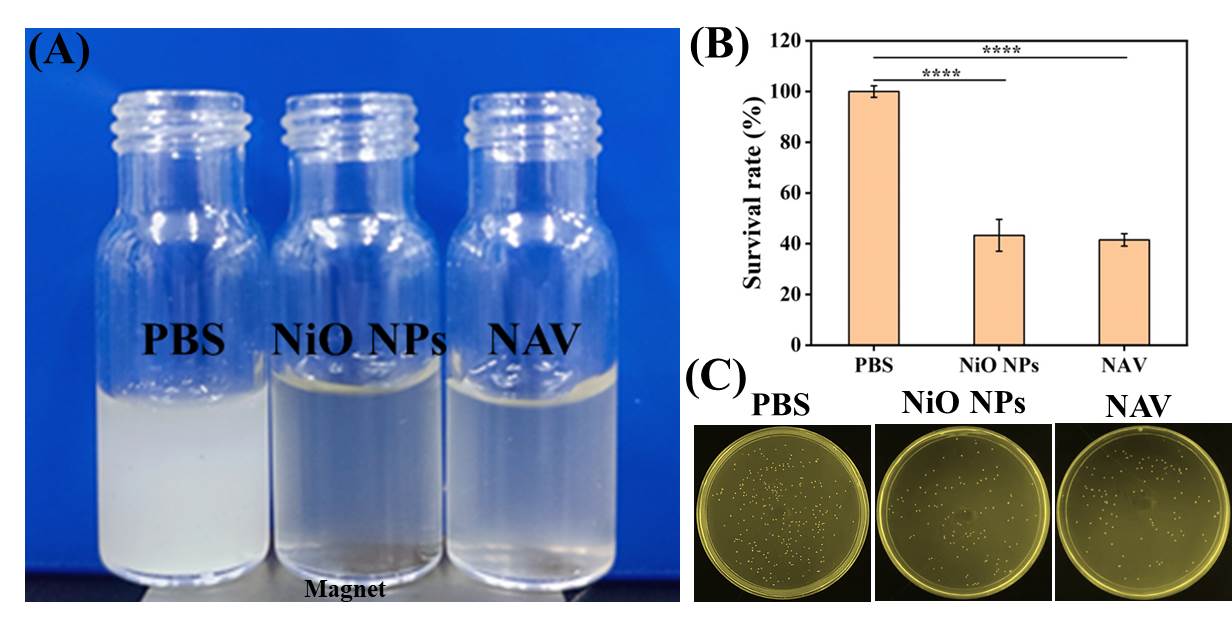


**Fig. S4** Antibacterial activity and binding ability of NiO NPs and NAV. (A) Binding of MRSA by NiO NPs and NAV. (B) The survival rate of MRSA after treatment with NiO NPs and NAV. (C) Photographs of colony formation of MRSA corresponding to (B).


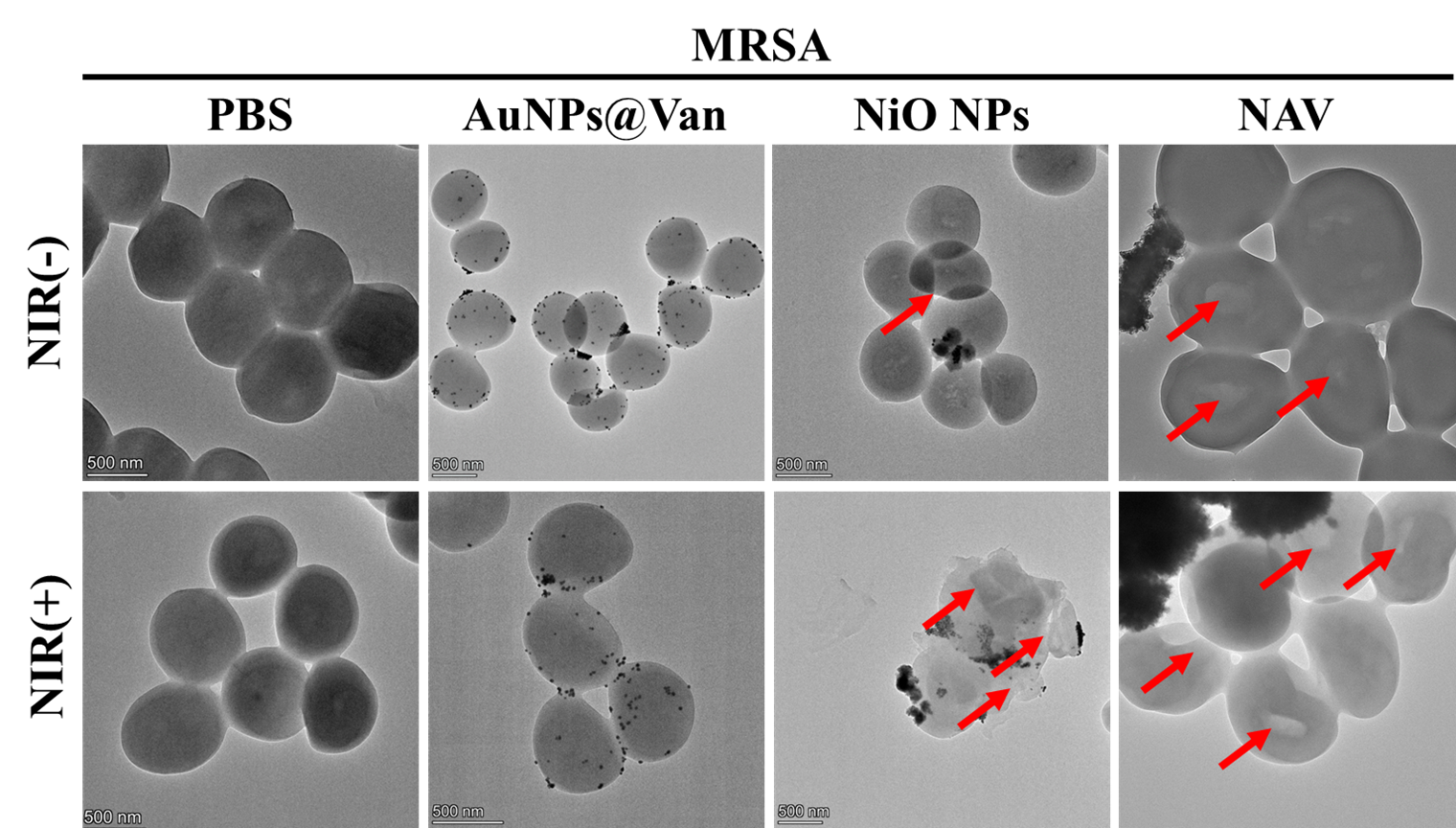


**Fig. S5** Morphologies of MRSA treated separately by PBS-NIR_(-)_, PBS-NIR_(+)_, AuNPs@Van-NIR_(-)_, NiO NPs-NIR_(-)_, NiO NPs -NIR_(+)_, NAV-NIR_(-)_ and NAV-NIR_(+)_ at 125 μg/mL with (NIR_(+)_) or without (NIR_(-)_) 808 nm NIR irradiation for 10 min.


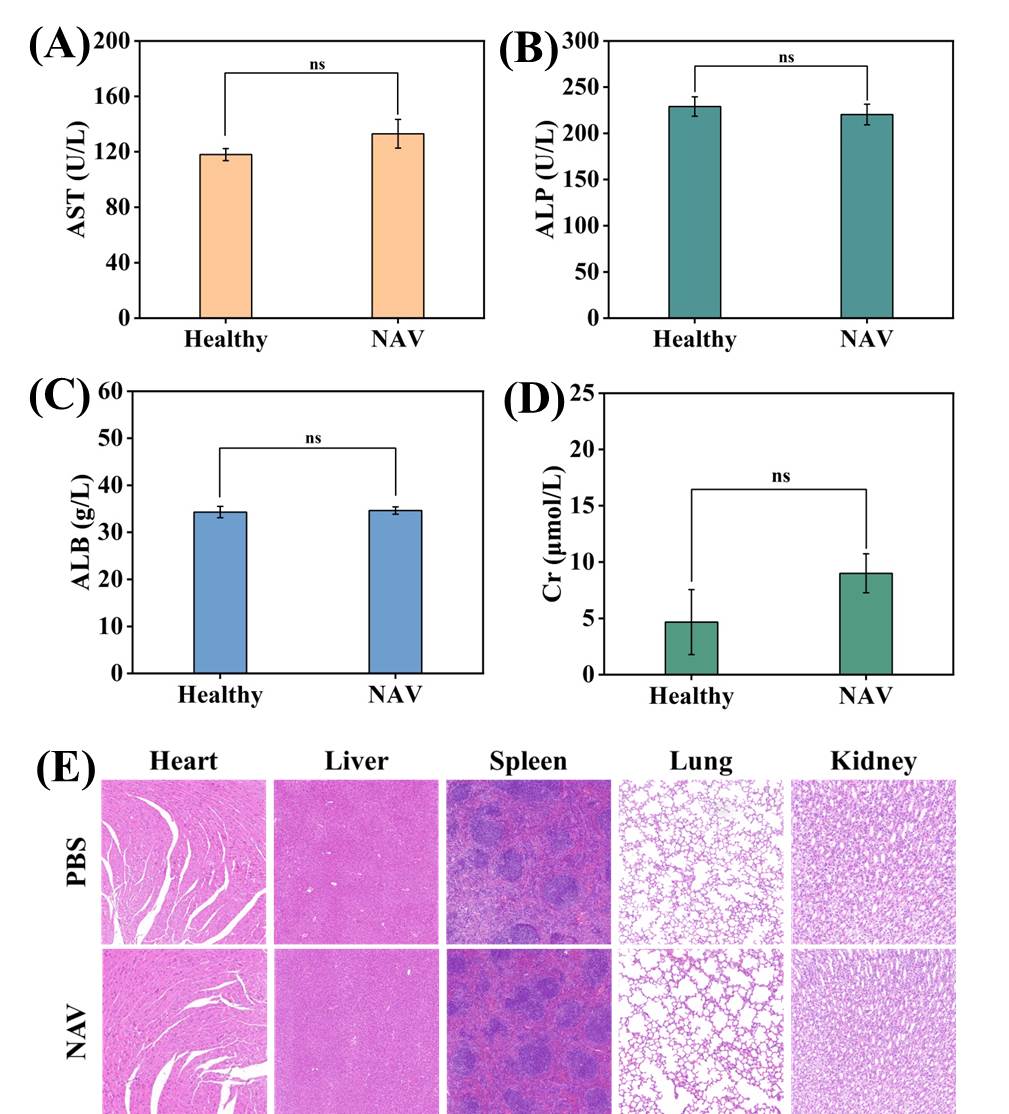


**Fig. S6** The toxicity assay of NAV *in vivo*. Changes of major serum biochemistry indicators of aspartate transaminase (AST) (A), alkaline phosphatase (ALP) (B), albumin (ALB) (C) and creatinine (D) in normal mice after being treated by PBS and NAV. (E) The H&E staining of internal organs (heart, liver, spleen, lung and kidney) after being treated by PBS and NAV.

**Table S2.** The wound healing rate of different treatment groups.

|  | PBS | PBS+NIR | NAV | NAV+NIR |
| --- | --- | --- | --- | --- |
| Healing rate | 72.2% | 79.9% | 82.6% | 93.5% |


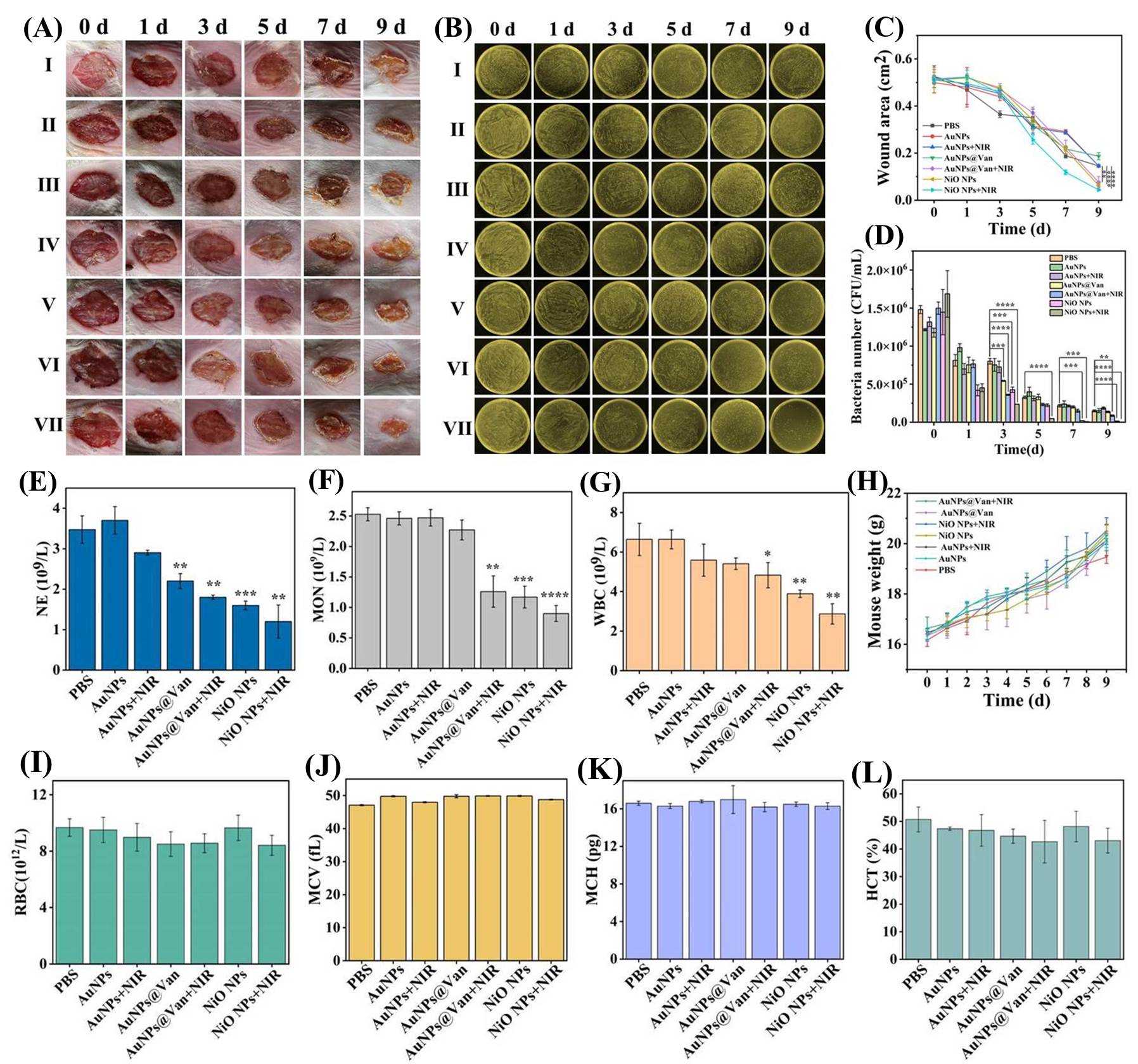


**Fig. S7** (A) Photographs of MRSA-infected skins treated with Ⅰ (PBS-NIR_(-)_), Ⅱ (AuNPs-NIR_(-)_), Ⅲ (AuNPs-NIR_(+)_), Ⅳ (AuNPs@Van-NIR_(-)_), Ⅴ(AuNPs@Van-NIR_(+)_),Ⅵ (NiO NPs-NIR_(-)_), and Ⅶ (NiO NPs-NIR_(+)_) after 0, 1, 3, 5, 7 and 9 days of therapy. (B) Corresponding agar plate experiment pictures of MRSA-infected skin in different treatment groups after 0, 1, 3, 5, 7 and 9 days of treatment. (C) Changes in the wound area of infected mice from day 0 to 9 during treatment. (D) The inhibition rate on bacterial growth for different treatment groups at different time intervals corresponding to (B). Changes of neutrophil (NE) (E), monocyte (MON) (F) and white blood cell (WBC) (G) levels in different treatment groups. (H) Changes in the body weight of MRSA-infected mice from day 0 to 9 during treatment. Changes of MRSA-infected mice in (I) red blood cells (RBC), (J) mean corpuscular volume (MCV), (K) mean corpuscular volume (MCH) and (L) hematocrit (HCT) after different treatments.


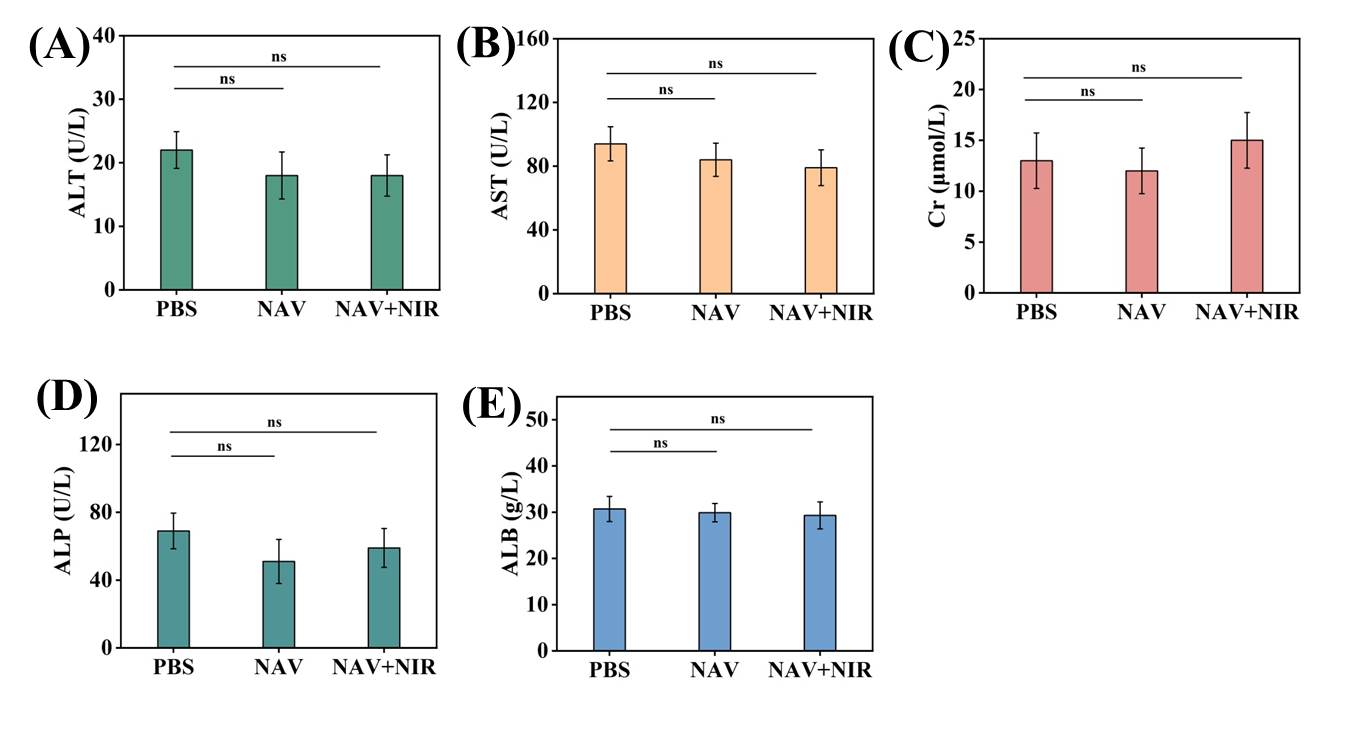


**Fig. S8** Changes of major serum biochemistry indicators of alanine transaminase (ALT) (A), aspartate transaminase (AST) (B), creatinine (C), alkaline phosphatase (ALP) (D), and albumin (ALB) (E) in MRSA-infected mice after 9 days of treatment.


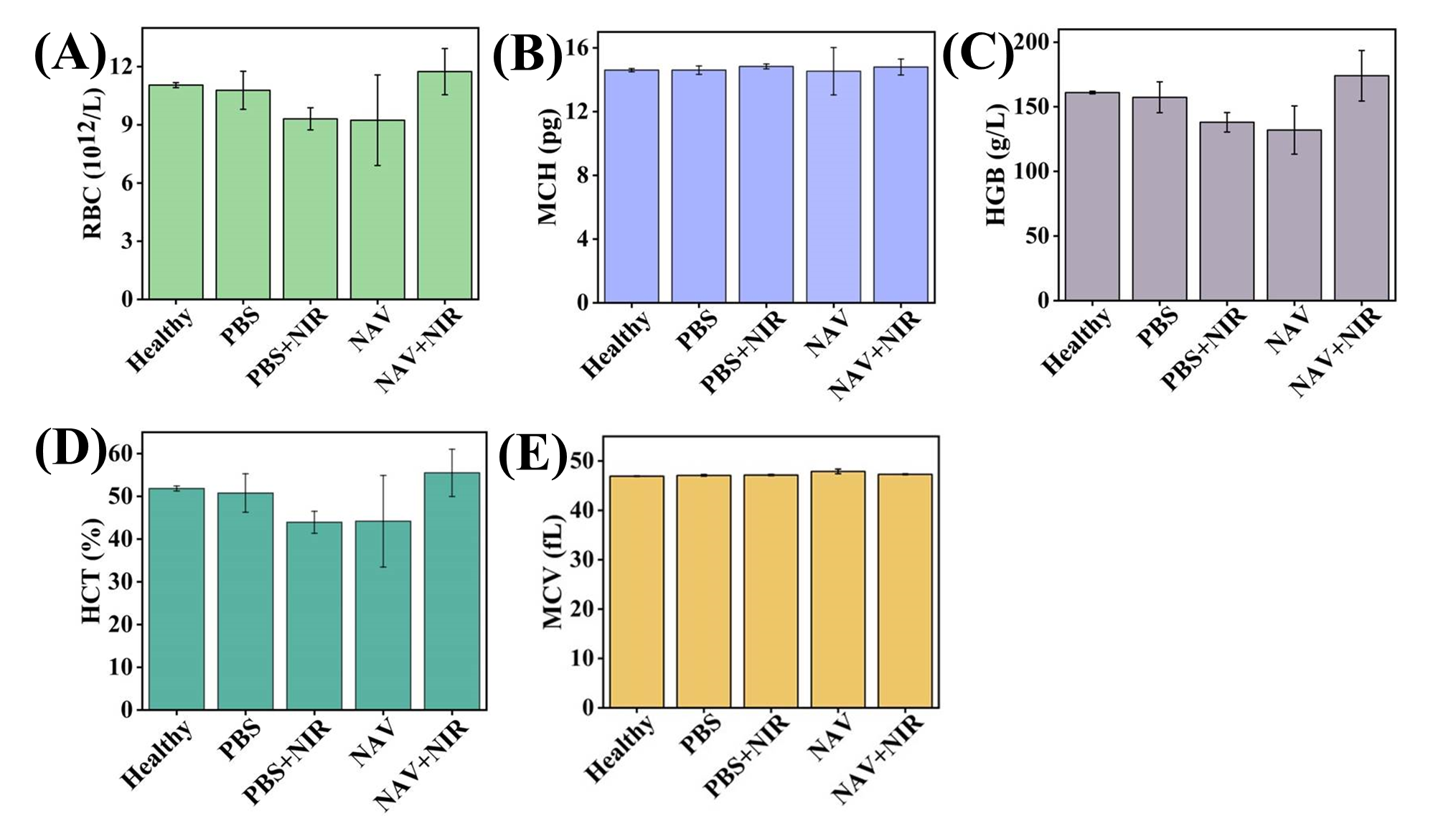


**Fig. S9** Changes of MRSA-infected mice in (A) red blood cells (RBC), (B) mean corpuscular volume (MCH), (C) hemoglobin (HGB), (D) hematocrit (HCT) and (E) mean corpuscular volume (MCV) after different treatments.


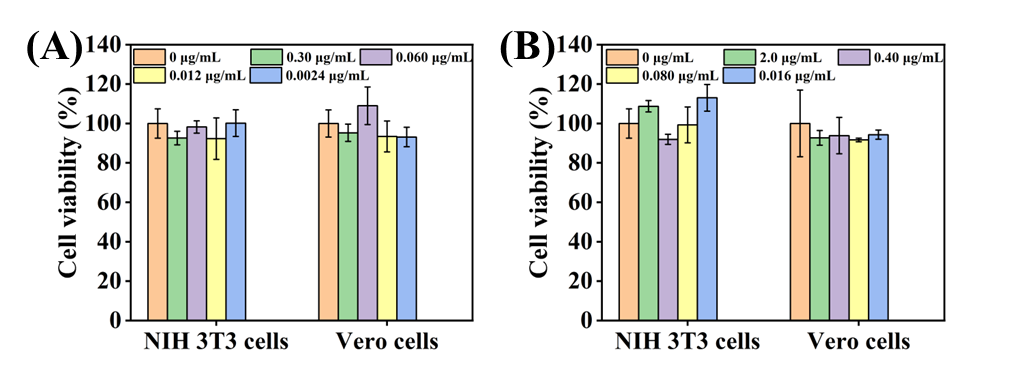


**Fig. S10** The viability of NIH 3T3 cells and Vero cells after 48 h of incubation with different concentrations of (A) Au^3+^ and (B) Ni^2+^.

1. Correspondence: [xjdu@tust.edu.cn](mailto:xjdu@tust.edu.cn) (X. Du), [wangshuo@nankai.edu.cn](mailto:wangshuo@nankai.edu.cn) (S. Wang).

   †These authors contributed equally. [↑](#footnote-ref-1)
